# Influence of nano-BN inclusion and mechanism involved on aluminium-copper alloy

**DOI:** 10.1038/s41598-024-56986-3

**Published:** 2024-03-16

**Authors:** Ziqi Zhang, Qi Zeng, Ning Wang, Lixia Wang, Quan Wu, Xin Li, Jiao Tang, Rong Li

**Affiliations:** 1 School of Mechanical and Electrical Engineering, Guizhou Normal University, Guiyang, China; 2 Guiyang Huaheng Mechanical Manufacture Co., Ltd, Guiyang, China

**Keywords:** Mechanical properties, Metals and alloys, Atomistic models, Electronic structure

## Abstract

Taking advantage of the high specific surface area of the nanoparticles, boron nitride (BN) nanoparticles were incorporated into the semi-solidified aluminium-copper alloy Al–5Cu–Mn (ZL201) system during the casting process, and its properties and enhancement mechanism were studied. The results shown that the BN in the new composite material is more uniformly distributed in the second phase (Al_2_Cu), which can promote grain refinement and enhance the bonding with the aluminium-based interface, and the formation of stable phases such as AlB_2_, AlN, CuN, etc. makes the tensile strength and hardness of the material to be significantly improved (8.5%, 10.2%, respectively). The mechanism of the action of BN in Al_2_Cu was analyzed by establishing an atomic model and after calculation: BN can undergo strong adsorption on the surface of Al_2_Cu (0 0 1), and the adsorption energy is lower at the bridge sites on the two cut-off surfaces, which makes the binding of BN to the aluminum base more stable. The charge transfer between B, N and each atom of the matrix can promote the formation of strong covalent bonds Al–N, Cu–N and Al–B bonds, which can increase the dislocation density and hinder the grain boundary slip within the alloy.

## Introduction

Nanoceramic particles of boron nitride are an effective reinforcing material for aluminium alloys due to their extreme hardness, wear resistance, and elevated-temperature stability^1–3^, which is of great significance for the modification and application of aluminium alloys. Previous studies had mainly analyzed the modification effect of ceramic particles of boron nitride on composites through experiments, and the results mainly focus on the preparation method, mechanical properties, and the content and dispersion of nanoparticles^4–7^. For example, L. Tharanikumar et al.^8–11^ investigated the effect of the addition of boron nitride particles with different volume fractions on the mechanical properties of aluminium alloys. The results showed that proper addition of boron nitride particles can significantly increase the strength and hardness of aluminium alloys while maintaining excellent elongation. The adaptation and influence of BN particles in different material fields and applications are diverse. Karabacak et al.^12,13^ found that adding BN particles to Al2024 and AA7075 matrix not only improves both hardness, but also enhances the transport performance of metal ions. This enhanced electrodynamic effect is helpful to adjust the transport behavior of metal ions and form a lubricating BN ternary film, which can effectively reduce the plastic deformation and wear of materials and improve the durability of materials. In addition, BN also plays an important role in Li-metal batteries. Liu et al.^14,15^ confirmed the effectiveness of BN in forming a protective layer during Li deposition and preventing branch growth through in-situ observation under optical microscope. At the same time, the electrochemical test results showed that the Li-metal battery with BN coating shows better performance and stability, which is helpful to improve the service life and safety of the battery. Moreover, BN also showed excellent efficiency in photo/electrocatalysis. According to the latest research, BN nanoparticles were beneficial for photocatalytic hydrogen production and gas sensing, promoted charge transfer at the interface of heterostructures, and significantly improved the catalytic activity in heterostructures^16,17^. Especially in some specific chemical reactions, the addition of these nanoparticles can significantly improve the catalytic efficiency, which opens up new opportunities for the development of photoelectrocatalysis^18^. In a word, BN particles play an important role in various material fields and applications, which makes BN an important material in many fields and has wide application prospects.

In recent years, more and more researchers have been focusing on combining first-principles calculations with ceramic nanoparticle-modified aluminium alloys for their studies. In terms of research methodology, researchers reveal the physical properties and microstructure of materials on an atomic or molecular scale, and predict the mechanical properties and deformation mechanisms of materials^19–22^. Therefore, the study of the properties of aluminium alloys reinforced with nanoceramic particles using first-principle calculations can provide ideas and theoretical basis for experimental design and interpretation of results. For example, Han et al.^23–25^ investigated the surface energy, adhesion, interfacial energy, and electronic structure of Ti/Al_3_Ti based on first principles. The results show that Al_3_Ti particles have excellent interfacial compatibility, which enables them to effectively limit the dislocation activities at the grain boundaries and within the crystals of aluminium alloys, thus improving the properties of the materials. Similarly, Ma et al.^26,27^ investigated the dispersion behavior of boron nitride particles in aluminium alloys and their limiting effect on the dislocation motion at grain boundaries through first-principles calculations and experimental tests. The results showed that boron nitride particles have a significant effect on the grain boundary strengthening of aluminium alloy, which can significantly improve the strength and hardness of the material. The above studies provide an essential reference for the properties of aluminium alloys reinforced with nanoceramic particles of boron nitride.

In addition, there have been numerous studies related to the microscopic mechanism of action of ceramic nanoparticles in Al matrix composites. It mainly includes the deposition law of nanoparticles in casting aluminium alloy, the effect on the organizational structure and mechanical properties of aluminium alloy, etc.^7,28–32^. ​However, current research in these areas still needs to improve on coarse grains, inhomogeneous phase transitions, interfacial interaction mechanisms that still need to be sufficiently clear, aggregation behavior of ceramic particles, and poor wettability in aluminium liquids^33^. The presence of Zn, Fe, Zr, and additional elements in the aluminium alloy melt also makes the hardness and wear resistance of the alloy decrease^34,35^. In response to the above problems, some scholars have found that the addition of ceramic-like particles to aluminium alloys can significantly improve the distribution of the second phase in the alloy and improve the performance of aluminium matrix composites. For example, Zhang et al.^36–38^ investigated the refinement of the as-cast organization of Al–Mg-Zn alloys in relation to the number density of Al_3_ (Sc, Zr) grains and the critical nucleation work of the grains, and discussed theoretically the enthalpies of formation and interfacial stability of the different ceramic particles, such as Al_3_ (Sc, Zr), by using first-principles calculations. B. Nemutlu et al.^39–41^ improved the wettability of boron nitride nanosheets, boron nitride nanotubes and other boron nitride materials with different morphologies by uniformly distributing them in an aluminium liquid to improve the properties of composites. Ma et al.^42,43^ investigated the mode of interfacial interaction of BN and TiC nanoceramic particles with aluminium alloys by first-principle calculations and experimental means simultaneously. It was shown that the interface can effectively suppress grain boundary slip and improve the strength and hardness of the composite to a certain extent. In addition, the formation of AlB_2_ and AlN phases in Al/BN composites has an important impact on the properties of the materials. Kvashnin et al.^2,44–46^ used high-angle dark field scanning transmission electron microscopy (HAADF-STEM) and energy dispersive X-ray spectroscopy (EDXS) to study Al/BN samples and found that the formation of AlB_2_ phase was driven by the diffusion of B atoms in the Al matrix, which was mainly located inside the Al grain, while the formation of the AlN phase was controlled by the diffusion of N atoms along the Al grain interface, and the thin layer was precipitated along the Al grain interface. Among them, the AlB_2_ phase has good mechanical properties and thermal stability, which can improve the strength and high-temperature performance of the composites. The AlN phase has high hardness and chemical stability, which can improve the hardness and wear resistance of composites. However, thus far, there are fewer reports on the adsorption and mode of action between the second phase and the reinforcing particles at the atomic scale for the incorporation of ceramic particles of boron nitride into cast aluminium-copper alloys, as well as the reasons for the enhancement of the stability and mechanical properties of the composites.

In this study, the Al–5Cu–Mn–0.5BN alloy specimens were firstly prepared by casting method, and their mechanical properties and micro-morphology were tested and characterized. Secondly, an attempt was made to model the surface of BN adsorption of Al_2_Cu (0 0 1) using a first-principles approach to simulate the shift of system energy before and after BN adsorption and to analyze the influence and mode of action of BN on the second phase Al_2_Cu. The electronic structures (energy band structure, density of states, differential charge density) with lower adsorption energies in the model are also calculated to analyze the stability of the material structure as well as the bonding state and bonding between the elements, and to explore the enhancement mechanism of the properties of the composites by the nanoceramic particles of boron nitride at the atomic level.

## Materials and methods

### Experimental methods

**Figure 1:**
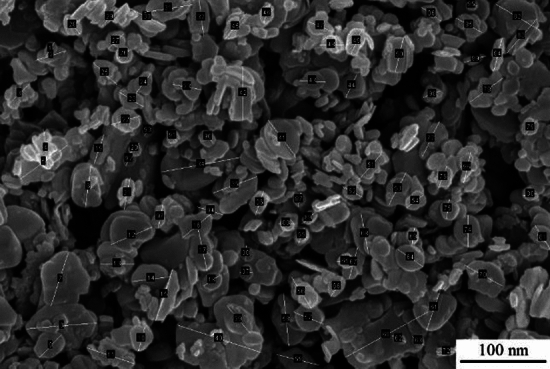
SEM image of nanoceramic particles of hexagonal boron nitride.

**Figure 2:**
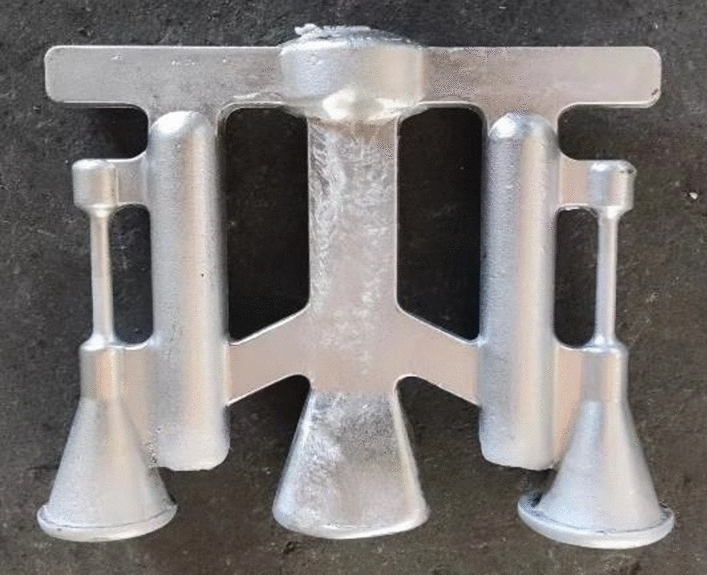
Foundry goods.

**Figure 3:**
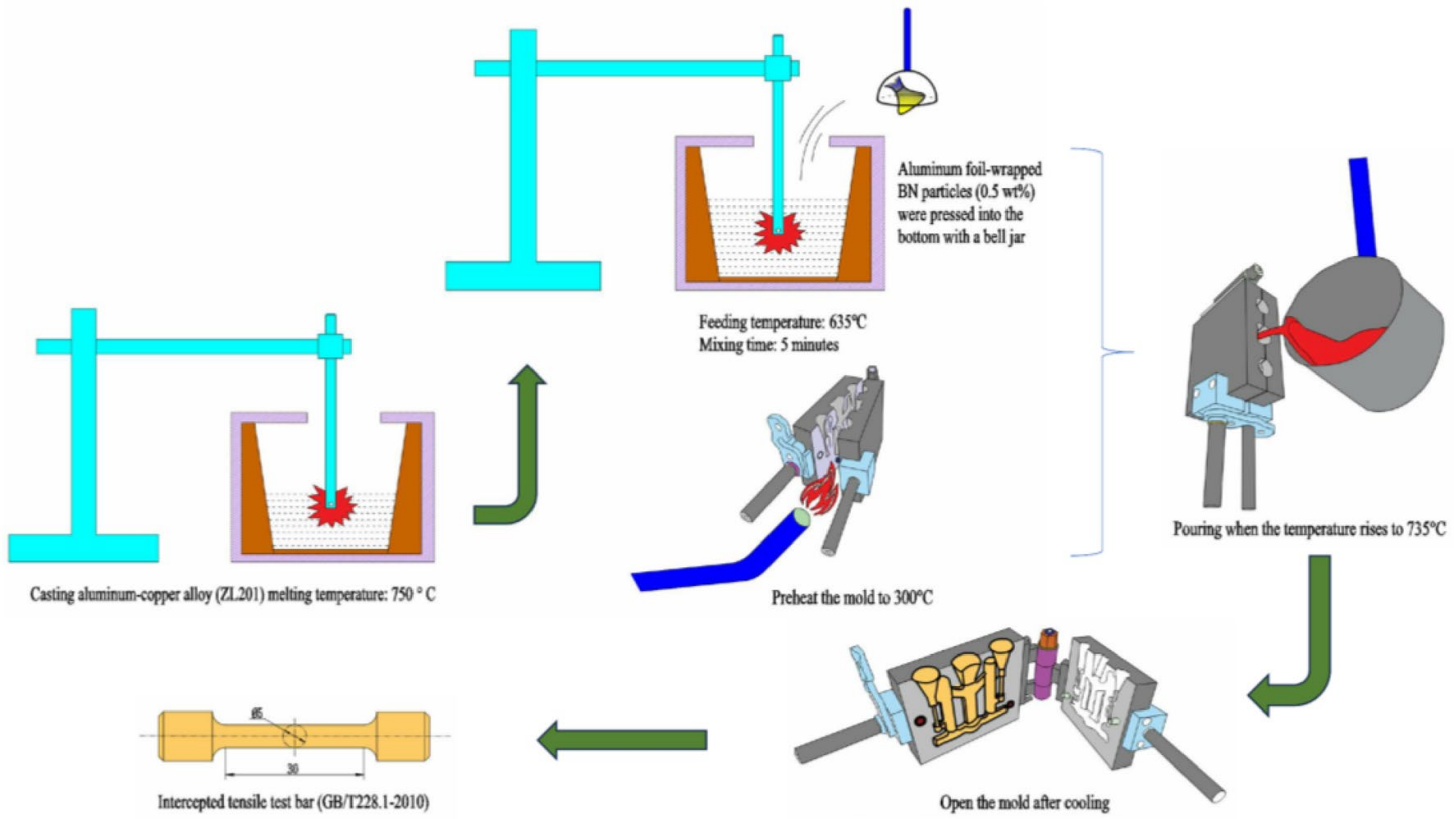
Schematic diagram of the sample casting process.

In this study, an Al–5Cu–Mn aluminium-copper alloy (ZL201) was used as the raw material, and its chemical elemental composition was shown in Table 1. The reinforcement is hexagonal boron nitride (hBN) with an average particle size of 100 nm and a purity of 99.9ω%, and its microscopic morphology is shown in Fig. 1, which is formulated according to a specific ratio so that the mass fraction of BN in Al–5Cu–Mn before the experiment is 0.5ω%. The casting method used in this experiment is metal-type casting; the mold material is cast iron, and graphite powder is used as a coating on the surface of its cavity to prevent the casting from sticking to the mold. First, the original alloy material was melted in a crucible at 750 °C, and then the temperature of the melt was reduced to 635 °C. Nanoceramic particles of boron nitride wrapped in aluminium foil were pressed into the bottom of the melt with bellows and stirred for 5 min. After the boron nitride particles were uniformly dispersed in the matrix, the melt was heated to 735 °C, salvaged to remove surface oxides and dross, and poured into a preheated 300 °C mold. After opening the mold, the casting (Fig. 2) was cooled at room temperature. The tensile test bar (*F* condition) required for the experiment and conforming to the national standard *GB/T228.1-2010* was intercepted, and the whole material preparation process is shown in Fig. 3. In order to reduce the error brought about by the number of experiments, as well as between the castings from different mold cycles due to the pouring process, the temperature inside the mold gradually increased to affect the fluidity of the melt, so that the castings exist in the production of performance differences. Therefore, five groups of different batches of castings were taken from each of the composites and master alloys for this experiment to intercept the test bars. Finally, the mechanical properties of the materials were measured using a tensile tester, a multifunctional hardness tester, and an electronic extensometer; the fracture morphology of the tensile specimens and the surface scanning of the chemical elements were carried out using a Sigma *300* scanning electron microscope (SEM) equipped with an X-ray spectrometer; the electron backscatter diffraction (EBSD) technique was used to obtain information on the size, shape, orientation, and grain boundaries of the crystals in the specimens; The microstructures of the materials were characterized using a transmission electron microscope (TEM), which also allowed us to observe the morphology and distribution of the strengthening phases.

**Table 1:** Al–5Cu–Mn aluminium–copper alloy (ZL201) chemical element composition and content (ω%).

Elements	Cu	Mn	Si	Zn	Fe	Zr	Al
Content	5.3	0.95	0.3	0.2	0.25	0.2	Allowance

### Adsorption modeling and calculation methods

**Figure 4:**
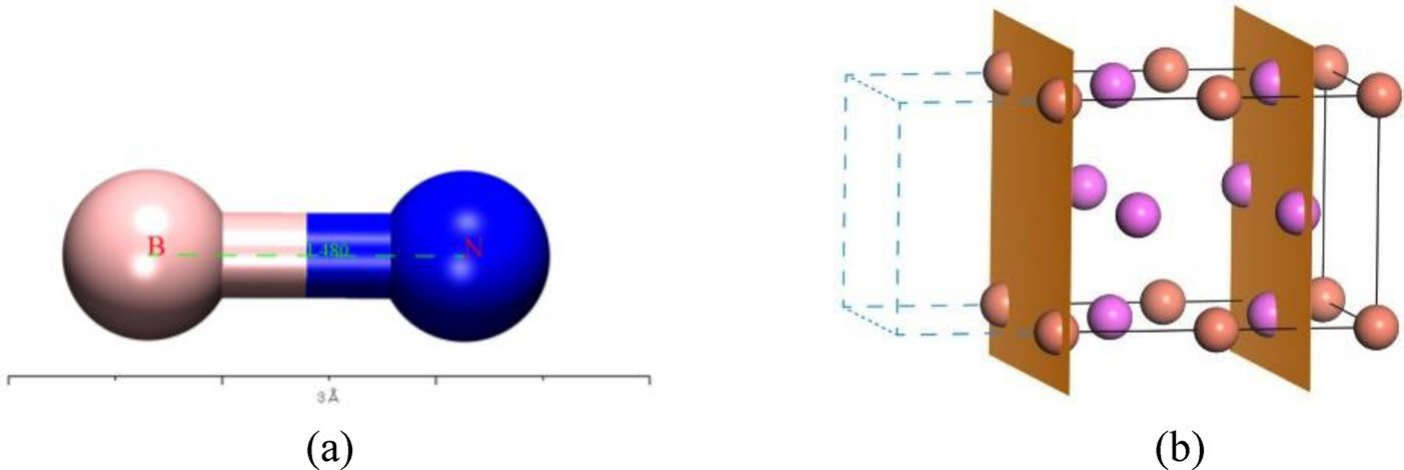
Adsorption model: (**a**) Adsorbent BN molecule; (**b**) Adsorption substrate Al_2_Cu (0 0 1) axial growth surface.

**Figure 5:**
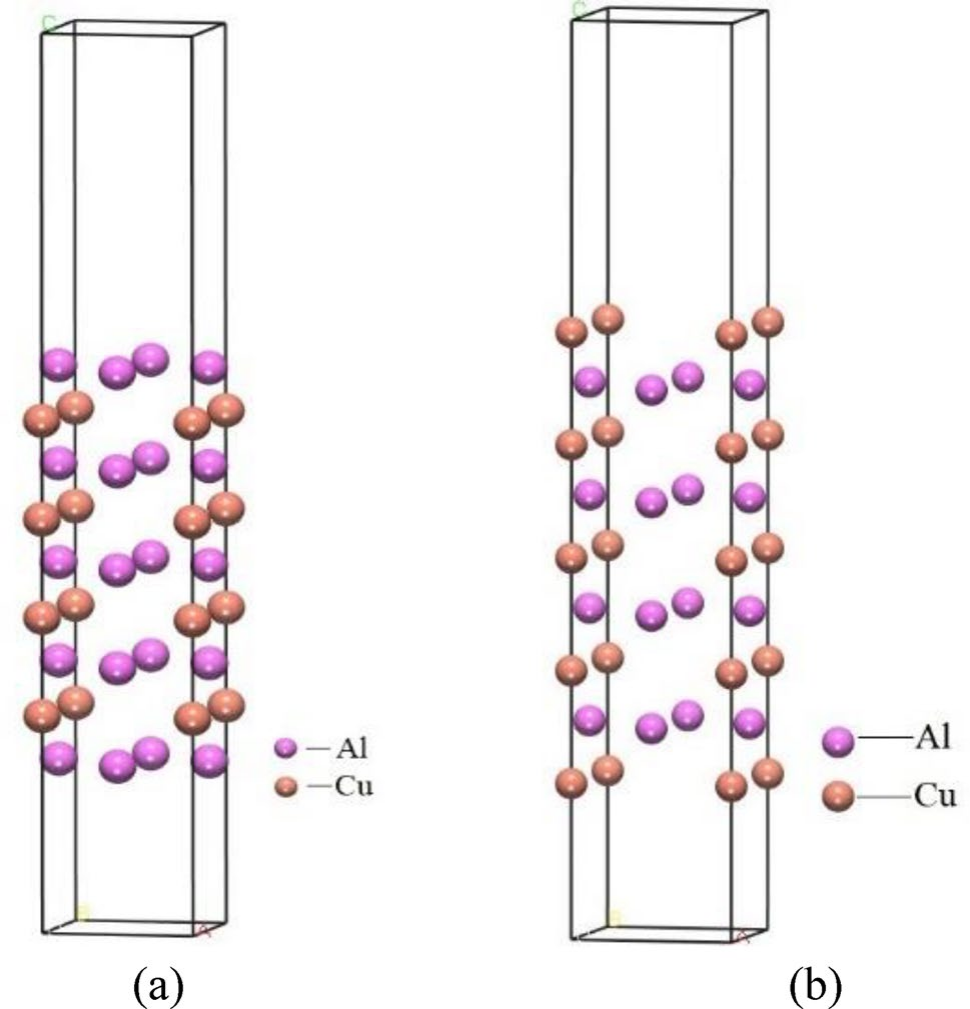
Al_2_Cu(0 0 1) surface modeling: (**a**) Al–Al_2_Cu (0 0 1) surface; (**b**) Cu–Al_2_Cu (0 0 1) surface.

In order to investigate the adsorption of BN by Al_2_Cu in Al–5Cu–Mn–0.5BN alloys, the simulation and computational model used for this study was constructed using the Visualizer module of the Materials Studio software. Due to the solidification properties of intermetallic compounds, the incipient Al_2_Cu phase dendrite grows axially and uniformly along its (0 0 1) direction^47^, so the Al_2_Cu (0 0 1) axial growth surface was selected as the adsorption surface as shown in Fig. 4. The adsorbent (BN) and adsorption substrate (Al_2_Cu) of the adsorption model are shown in Fig. 4-a,-b, respectively. The Al_2_Cu space group is *P*1 and the lattice constants are: a = b = 4.10518 Å, c = 2.89131 Å, *α* = *β* = *γ* = 90°, and *V* = 48.7259Å^3^. The Al_2_Cu (0 0 1) surface is a polar surface, and the Al_2_Cu (0 0 1) surface can be categorized into two cutoffs, Al–Al_2_Cu and Cu-Al_2_Cu, according to the different types of atoms on its polar surface, as shown in Fig. 5-a,-b.

**Figure 6:**
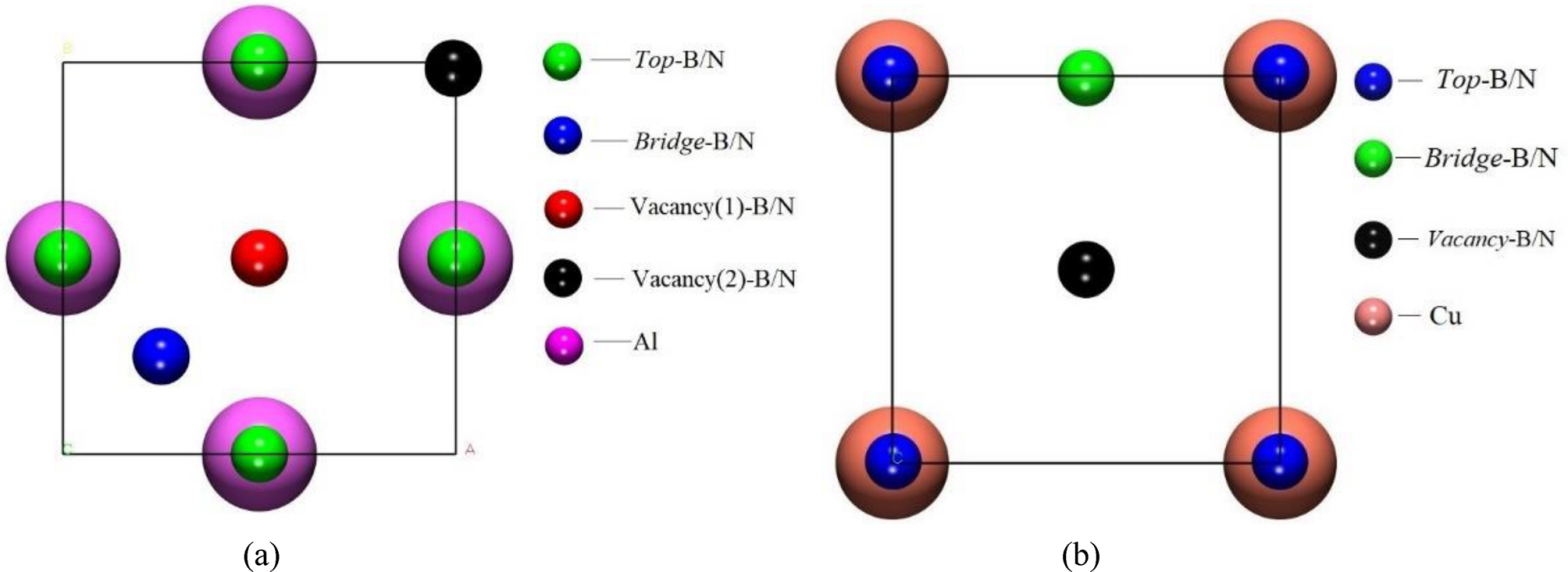
BN adsorption position: (**a**) Location of B/N adsorption on the Al–Al_2_Cu (0 0 1) surface; (**b**) Location of B/N adsorption on the Cu–Al_2_Cu (0 0 1) surface.

In order to compare the adsorption states of different atoms, two kinds of atoms in BN were adsorbed in the Al_2_Cu (0 0 1) surface model, respectively, and the adsorption positions are shown in Fig. 6. Among them, the highly symmetric adsorption positions of the Al–Al_2_Cu (0 0 1) surface model are Top, Bridge, Vacancy 1 and Vacancy 2, as shown in Fig. 6-a. The highly symmetric adsorption positions for the Cu–Al_2_Cu (0 0 1) surface model are Top, Bridge and Vacancy, as shown in Fig. 6-b. Smaller atoms represent different adsorption positions of B and N atoms on the Al_2_Cu (0 0 1) surface, and larger atoms represent adsorption surface atoms. Based on the above, a total of 14 adsorption configurations of BN adsorbed on the Al_2_Cu (0 0 1) surface were established. The number of atomic layers in the adsorption substrate for each configuration was taken to be 9, the vacuum layer thickness was taken to be 18 Å, 9 Å above, and 9 Å above and below, and the Al_2_Cu (0 0 1) surface model was modeled with three layers fixed in the middle and three layers above and below for surface chirp. The B-Al bond length was taken to be 1.95 Å, the B-Cu bond length to be 2.05 Å, the N-Al bond length to be 1.75 Å and the N-Cu bond length to be 1.85 Å. The B-Al bond length was taken to be 1.95 Å and the B-Cu bond length to be 2.05 Å.

Based on the first-principles approach under density-functional theory (DFT), this study performs all the simulation calculations through the VASP 5.4.4 (Vienna Ab-initio Simulation Package) software package^48,49^. For the interaction between nuclei and valence electrons in the alloy system is accurately described by the projective affixed wave method (PAW), the electronic wave function is expanded by means of a plane-wave basis group, and the exchange–correlation energy between the electrons is treated by the PBE generalized treatment under the generalized gradient approximation (GGA)^50,51^. The *K*-points in the Brillouin zone are generated using a grid centered at the Gamma point. The electronic grouping of the valence layer outside the nucleus of each atom is Al–3*s*^2^3*p*^1^, Cu-3*d*^10^4*s*^1^, B-2*s*^2^2*p*^1^, N-2*s*^2^2*p*^3^. After convergence tests, the plane wave truncation energy is determined to be 600 eV, and the *K*-point is taken to be 10 × 10 × 1. The Kohn–Sham equations are solved using the self-concordant loop (SCF). The energy convergence value is set to be 1 × 10^–5^ eV/atom, and the force convergence is controlled to be within –0.01 eV/nm.

For the simulations, the established adsorption configuration is first structurally optimized so that the crystalline cell is in the ground state. After the optimization, energy calculations were performed for the adsorbents and adsorbing substrate as well as the assemblage, respectively, to compute the interactions between BN and the Al_2_Cu (0 0 1) surface, and then the adsorption energy of BN on the Al_2_Cu (0 0 1) surface was derived according to Eq. (1)^52,53^.

1 $${\text{E}}_{{{\text{ad}}}} = {\text{E}}_{{{\text{BN}}/{\text{Al2Cu }}\left( {0 \, 0{ 1}} \right)}} - {\text{n}}_{{{\text{BN}}}} {\text{E}}_{{{\text{BN}}}} - {\text{E}}_{{{\text{Al2Cu }}\left( { \, 0 \, 0{ 1}} \right)}}$$

In Eq. (1), E_ad_ is the adsorption energy, $\text E_{\text{BN}/\text{Al}_2\text {Cu} \,\left( {0 \, 0{ 1}} \right)}$ is the system energy of the surface model after adsorption, E_BN_ is the energy of the adsorbate BN alone, n_BN_ is the number of atoms in the adsorbate BN with a value of 2, and $\text E_{\text{Al}_2\text {Cu} \,\left( {0 \, 0{ 1}} \right)}$ is the energy of the surface model of adsorbed substrate Al_2_Cu (0 0 1). Secondly, the energy band structure, density of states and fractional density of states of the system are calculated using the optimized configuration and their electronic structures are explored. Finally, the differential charge densities of the adsorbed configurations are calculated to analyze the charge gain, loss, and transfer of BN on the Al_2_Cu (0 0 1) surface.

## Results and analysis

### Experimental results

#### Mechanical properties

**Figure 7:**
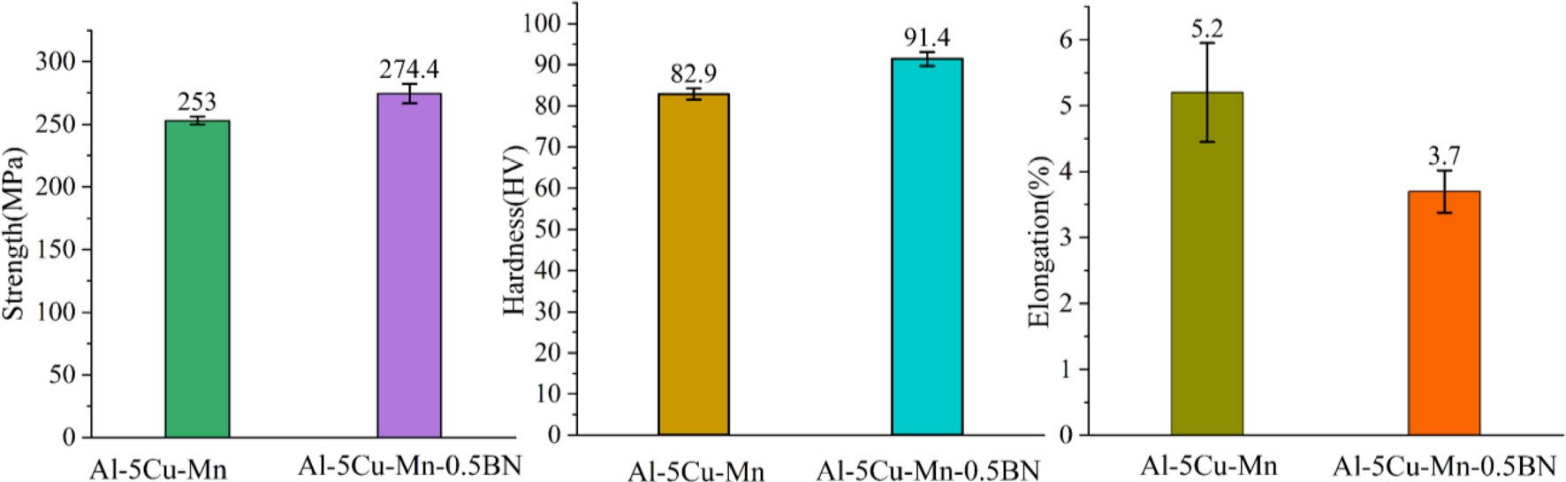
Discrete mechanical properties of alloys before and after BN addition.

Tables 2 and 3 showed the results and comparisons of the mechanical property tests of the two alloy materials under this experimental design. The test group number 1 − x represents the original alloy specimens, 2 −x represents the composite specimens. From the tables, they can be seen that after the addition of BN, the strength and hardness of the Al–5Cu–Mn–0.5BN alloy have been significantly increased, and the elongation have been reduced compared to that of the Al–5Cu–Mn alloy. Figure 7 showed the degree of discretization of each set of test data. Compared the mean results, it can be seen that the strength and hardness of the composites after the addition of BN are 274.4 MPa and 91.4 HV, which are improved by 8.5% and 10.2% (253 MPa and 82.9 HV), respectively, compared to the original alloy material. The elongation of the composite material has been reduced to 3.7%, which may be related to the fact that boron nitride itself has a demanding nature, which acts as a rigid reinforcement at the grain boundary and within the crystals, making it more difficult for the alloy to become ductile under load and suppressing the plastically of the whole composite material. The addition of Boron Nitride is beneficial in improving the mechanical properties of the cast aluminium alloy Al–5Cu–Mn, particularly in terms of strength and hardness, but at the expense of some elongation.

**Table 2:** Results of mechanical properties of composites before and after BN incorporation

Experimental group number	Mechanical properties		
	Strength (MPa)	Hardness (HV)	Elongation *Δ *(%)
1–1	254	83.5	5.2
1–2	257	84.6	4.0
1–3	251	81.9	5.6
1–4	255	83.8	5.1
1–5	248	80.8	6.3
2–1	269	91.4	3.9
2–2	283	92.9	3.5
2–3	262	88.2	4.3
2–4	277	91.9	3.4
2–5	281	92.6	3.7

**Table 3:** Mechanical properties of alloys before and after the addition of BN with standard deviation

Alloy materials	Mechanical properties		
	Strength (MPa)	Hardness (HV)	Elongation *Δ *(%)
Al–5Cu–Mn	253 ± 3.16	82.9 ± 1.38	5.2 ± 0.75
Al–5Cu–Mn–0.5BN	274.4 ± 7.84	91.4 ± 1.68	3.7 ± 0.32

To further compare the mechanical properties of the materials, the yield strength and Young's modulus of the two alloys were calculated from the stress–strain curves, and the results are shown in Table 4. It can be seen that the yield strength and Young's modulus of the Al–5Cu–Mn–0.5BN composite have been improved, indicating that the deformation resistance and stiffness of the material have been significantly improved after the addition of BN.

**Table 4:** Yield strength and Young's modulus of alloys before and after the addition of BN

Alloy materials	Yield strength *σ*_y_ (MPa)	Elastic modulus *E* (GPa)
Al–5Cu–Mn	183	34.35
Al–5Cu–Mn–0.5BN	215	43.84

#### Fracture morphology

**Figure 8:**
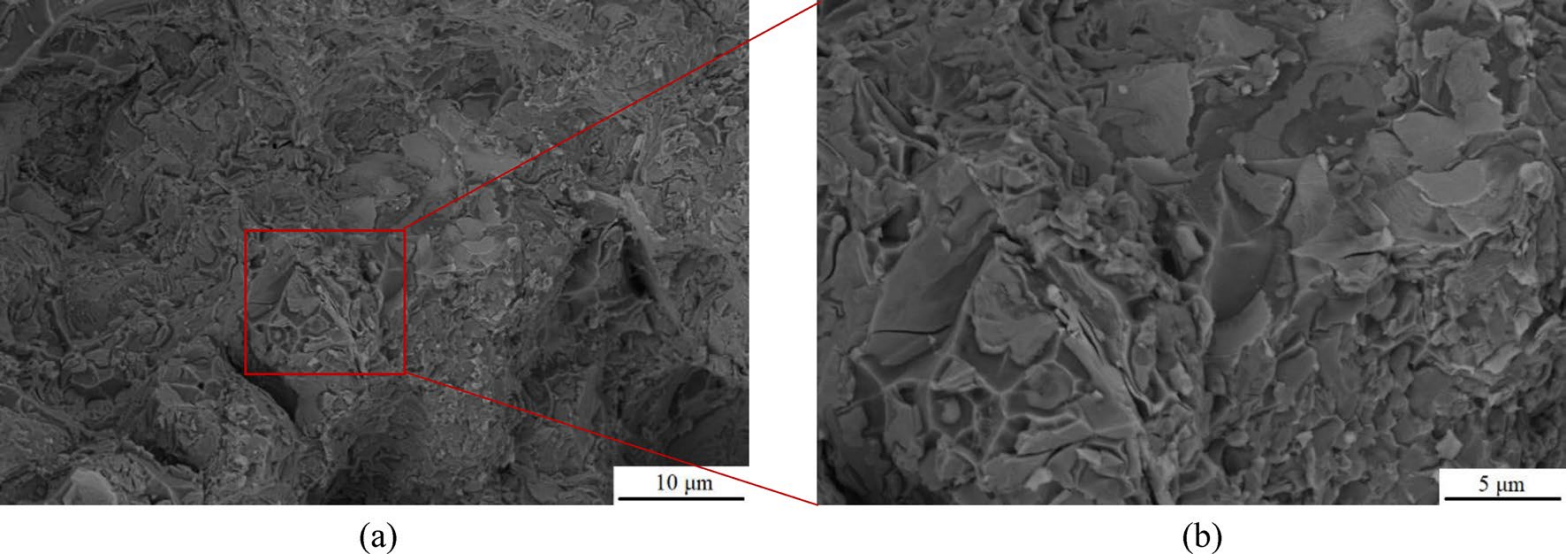
SEM image of fracture morphology of specimen 1–4: (**a**) Low magnification; (**b**) High magnification.

**Figure 9:**
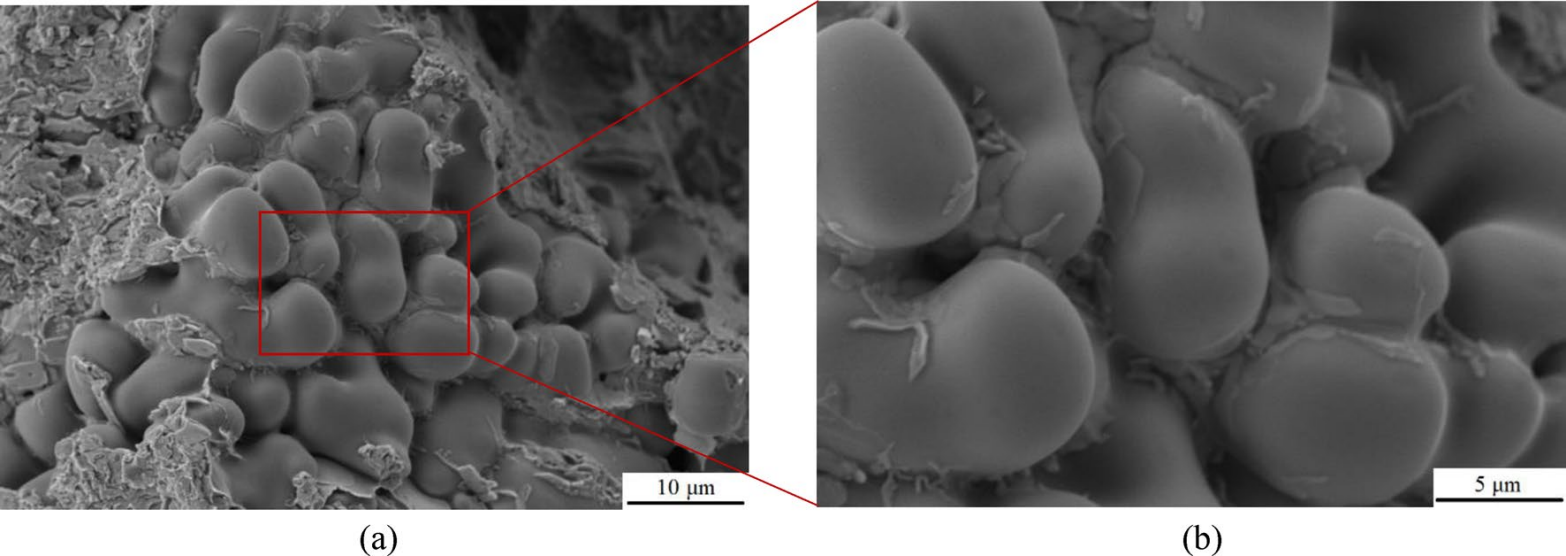
SEM image of fracture morphology of specimen 1–2: (**a**) Low magnification; (**b**) High magnification.

In order to compare the strengthening effect of BN particles on alloy materials, the fracture morphology of the two best groups of specimens (specimens 1–2, 1–4 and specimens 2–2, 2–5) for strength and hardness of alloy materials after stretching were photographed and analyzed. Figures 8 and 9 showed the fracture morphology of the specimens with the best strength and hardness in the raw material Al–5Cu–Mn alloy. It was observed that irregular tough nests and lamellar dendrites were present in the fracture morphology of specimen 1–2, and the alloy exhibited some plastic properties. The formation of lamellar dendritic tissue is related to the copper content and solidification rate of the material, due to the higher copper content in aluminium-copper alloys and the lower solidification rate compared to aluminium^54^. Therefore, during the cooling process, the copper-enriched region takes a longer time to solidify into a solid state, which makes it easy to form a lamellar dendritic organization. The structure of lamellar dendrites may lead to the deformation of grain boundaries, which affects the plastic deformation and fracture behavior of the material. The fracture morphology of specimen 1–4 showed smooth and rounded spherical features. It has been shown that non-wettable alumina can form non-uniform pores and heterogeneous vapor pores in aluminium liquids^55^, so the spherical morphology in this experiment may be due to the uneven volume expansion and contraction of the alloy during solidification, which produces air holes or pores in the interior. With the external stretching action makes the oxide inclusions encapsulated on the outside of the sphere extend and crack along the microcracks, subsequently exposing the spherical organization as shown in Fig. 9.

**Figure 10:**
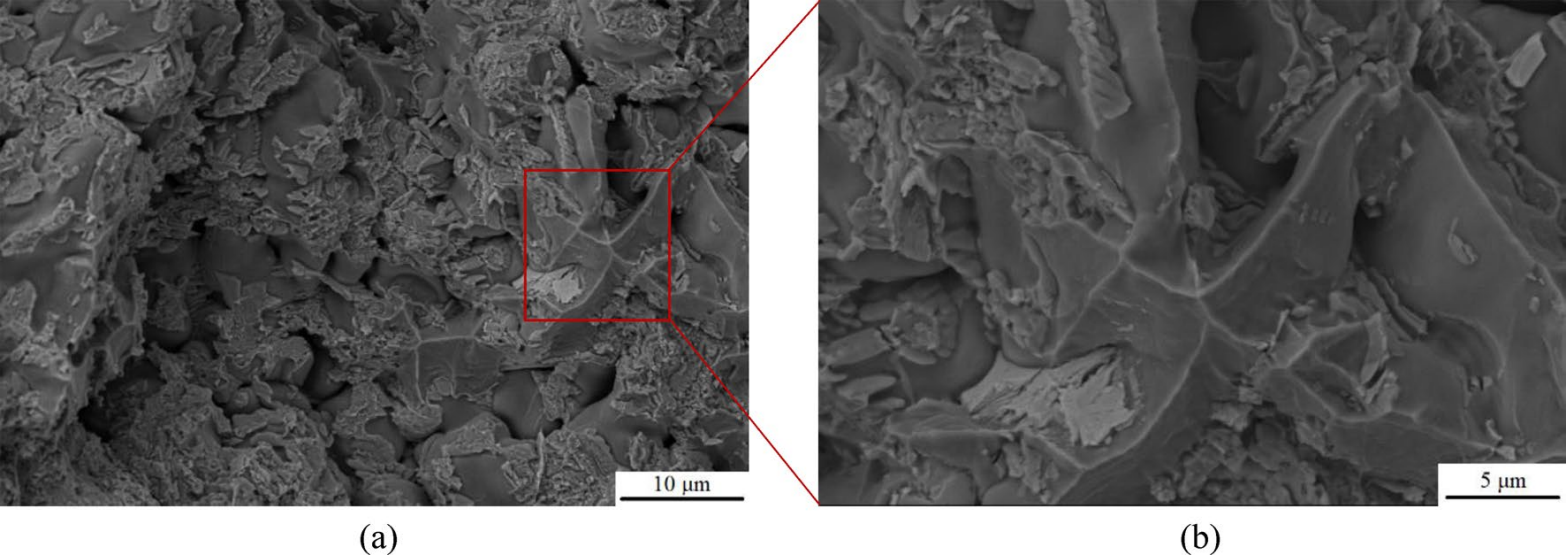
SEM image of fracture morphology of specimen 2–2: (**a**) Low magnification; (**b**) High magnification.

**Figure 11:**
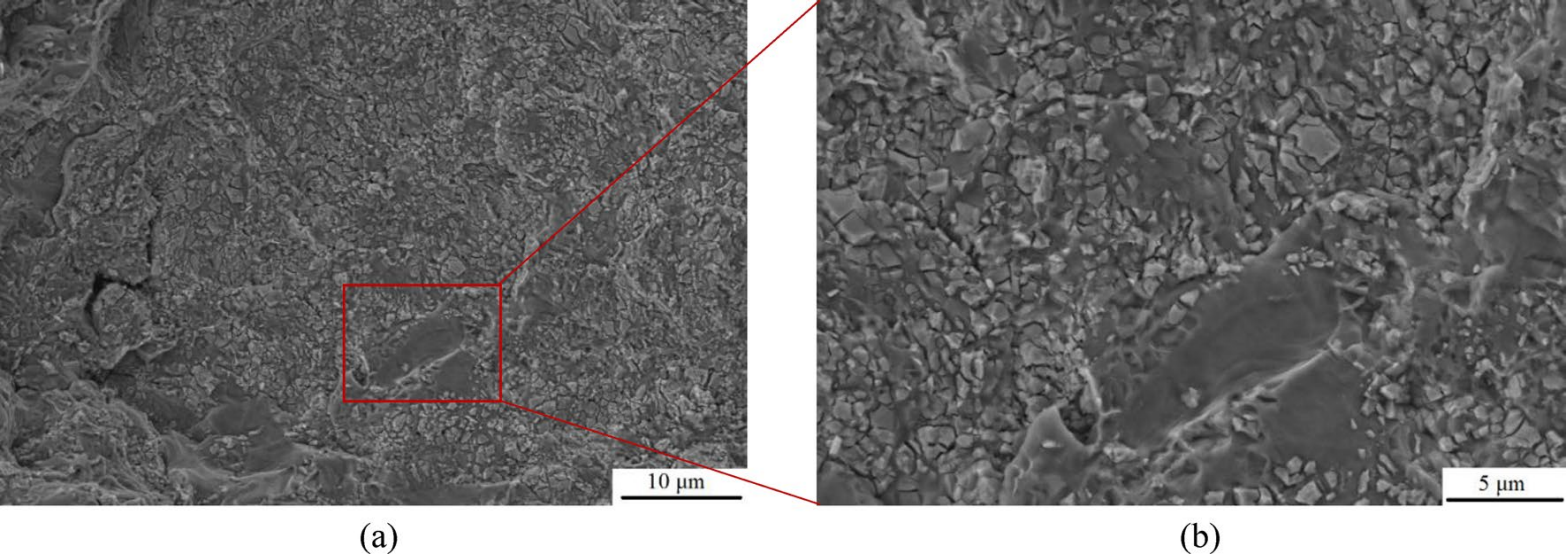
SEM image of fracture morphology of specimen 2–5: (**a**) Low magnification; (**b**) High magnification.

After the addition of 0.5ω% BN particles, the fracture morphology of the specimen changed significantly. As shown in Fig. 10, the fracture surface of specimen 2–2 exhibits a quasi-dissociative fracture with localized torn ribs and cliffs. The sharp angles of these torn ribs, and the steepness of the cliffs in the fine regions, result in an increase of the brittleness of the material. This fracture surface makes the crack path more complex and limits crack propagation, which contributes to the strength and hardness of the material. In contrast, the fracture surface of specimen 2–5 has numerous bright surfaces and particles present, as shown in Fig. 11-a, which could be either the second phase Al_2_Cu or the enhanced phase containing BN particles. Each bright surface in the fracture is the interface of a grain, and the morphology of each grain can be distinctly seen in high-magnification observations, similarly to the accumulation of ice-sugar cubes, which are gray-white in distribution as shown in Fig. 11-b. Some experiments have shown that this phenomenon is related to the refinement effect of boron nitride particles^56,57^, and the fine BN particles help to increase the number of grain boundaries, which makes the alloy's grain size reduced and uniformly dispersed, and improves the wettability of the molten state BN particles in the alloy to a certain extent. In order to determine the effect of the addition of BN on the grain size of the alloy material, electron backscatter diffraction (EBSD) was performed on the two test specimens, providing a better understanding of the microstructure and mechanical properties of the composites.

#### EBSD

**Figure 12:**
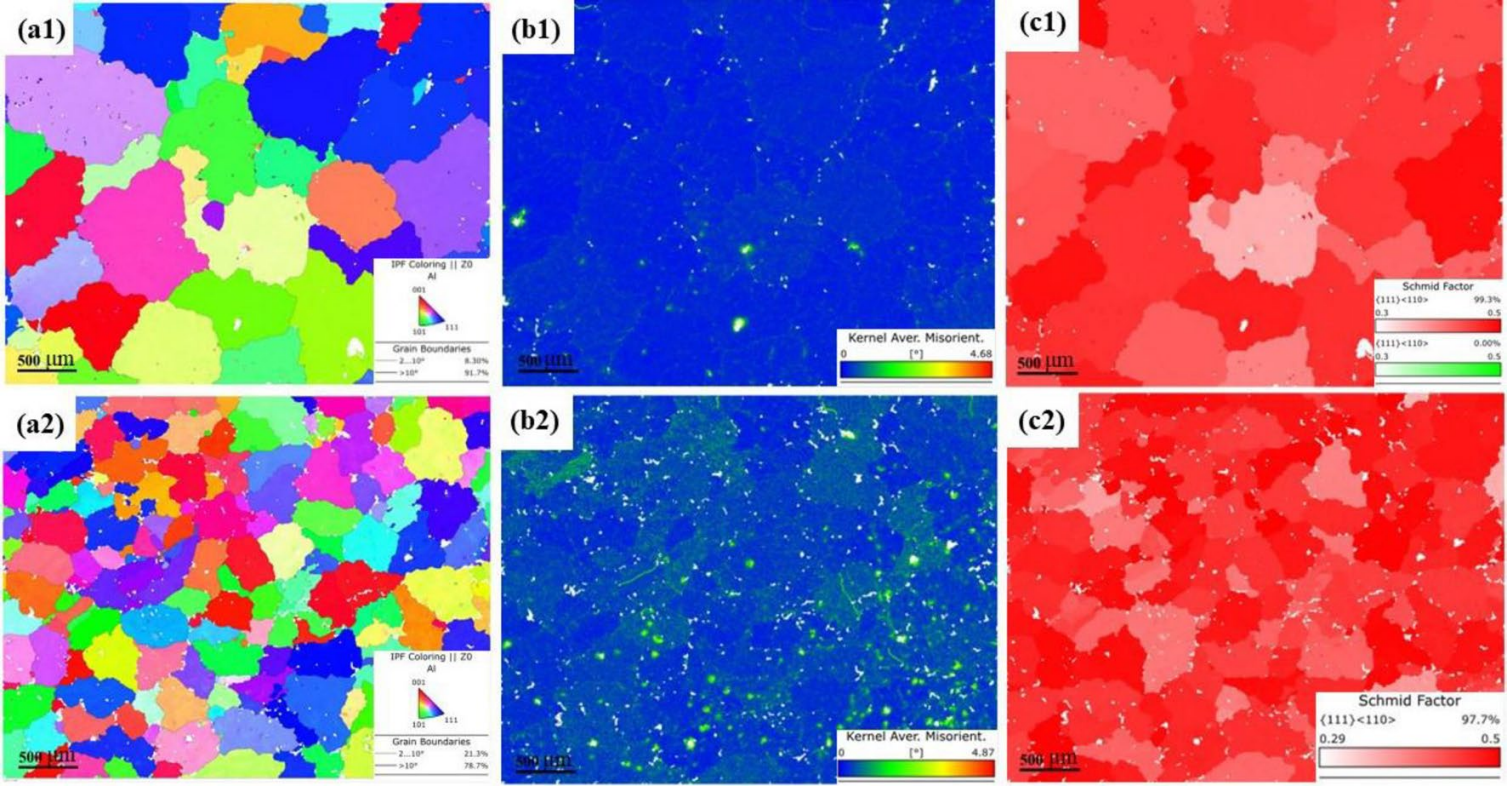
EBSD results of the materials before and after the addition of BN: (**a1**, **a2**) show the orientation plots of sample 2^#^ and sample 3^#^ ; (**b1**, **b2**) show the KAM distributions of sample 2^#^ and sample 3^#^; (**c1**, **c2**) show the Schmid factor plots of sample 3^#^ and sample 3^#^.

**Figure 13:**
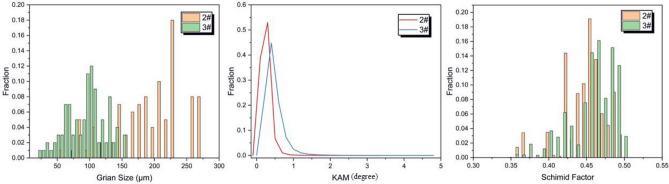
Statistical results of particle size, KAM, and Schmid factor for the two samples.

The orientation relationship and particle size between BN and Al are critical to the bond strength and have a significant effect on the strain or stress state during deformation, so the region near the fracture after the specimen has been stretched can be used as an EBSD specimen. Figure 12 show the EBSD (Electron et al.) results of two alloy specimens before and after BN incorporation, and Fig. 13 shows the statistical results of specimen grain size, KAM, and Schmid factor, respectively, where 2^#^ is an Al–5Cu–Mn specimen and 3^#^ is an Al–5Cu–Mn–0.5BN specimen. Based on the EBSD image results, information on grain size, crystallographic orientation, mismatch, and deformation behavior of the grains in the samples can be determined. Figure 12-a1,-a2 show the orientation maps of sample 2^#^ and sample 3^#^, where different colors represent different crystallographic orientations; Fig. 12-b1,-b2 show the KAM (mean core mismatch) distribution maps of sample 2^#^ and sample 3^#^. KAM values indicate the average mismatch between neighboring grains, with lower KAM values indicating a lower degree of mismatch, indicating good interfacial connections or fewer defects. Figure 12-c1,-c2 show the Schmid factor distribution maps for sample 2^#^ and sample3^#^, where the Schmid factor is a measure of the slip system in the crystal. Interfacial connection is good, or there are fewer defects; Fig. 12-c1,-c2 show the distribution of the Schmid factor for sample 2^#^ and sample 3^#^. The Schmid factor is a measure of the activity of the slip system in the crystal, which indicates the ease of deformation of the crystal under the applied stress, and the distribution of the Schmid factor provides information about the deformation behavior of the sample. From Fig. 13, it can be seen that the grain sizes of both samples are normally distributed, and both are equiaxial grains. However, compared with the 2^#^ sample, the grain size of the 3^#^ sample with BN added is significantly reduced (compare Fig. 12-a1,-a2), which indicates that the BN nanoparticles play a role in refining the grains inside the aluminium-copper alloy. The strength and hardness of the composites are improved. Regarding the Schmid factor, the slip system of both samples is unchanged, and there is no apparent weaving, so BN has little effect on the Schmid factor distribution of the original alloy material. However, the KAM value in the 3^#^ sample is slightly larger. The GND density is higher, which is due to a large number of BN grains agglomerating around the second phase Al_2_Cu. The material is prone to inhomogeneous deformation, causing the plastically to decrease, which is consistent with the test results of elongation in the previous tensile test.

### Adsorption and electronic structure property calculations

#### Adsorption energy

Tables 5 and 6 showed the system energies for different cutoffs and adsorption sites of BN adsorbed on the Al_2_Cu (0 0 1) surface, and the interaction modes can be derived from Eq. (2)^58,59^. As can be seen from Tables 5 and 6, the values of adsorption energy produced by BN adsorption at the 14 adsorption positions on the Al_2_Cu(0 0 1) surface are all negative, which indicates that BN produces stable chemical adsorption at all 14 highly symmetric adsorption positions on the Al_2_Cu (0 0 1) surface, whereas higher absolute values of the adsorption energy imply that the BN particles form a stronger bond with the surface of the substrate^60^. For the Al–Al_2_Cu (0 0 1) surface, the adsorption energies of the B atoms are less than − 6 eV at both the bridge (B) and top (T) sites, while the N atoms are less than − 6 eV at both the bridge (B) and vacancy (V) sites. Similarly, the adsorption energies of both atoms at "V1" when adsorbed at the vacancy (V) are smaller at − 4.46 eV and − 6.59 eV, respectively, whereas the adsorption energy of the system reaches a minimum when both atoms are adsorbed at the bridge (B). For the Cu–Al_2_Cu (0 0 1) surface, the adsorption energy of the B atoms is less than − 6 eV at the bridge site (B), whereas the N atoms are less than − 6 eV at the bridge site (B) and the vacancy site (V). Similarly, the adsorption energy of the system is minimized when both atoms are adsorbed at the bridge site (B).

2 $$\left\{ {\begin{array}{*{20}l} {{\text{E}}_{{{\text{ad}}}} > 0\, {\text{Repulsion}}} \hfill \\ {{\text{E}}_{{{\text{ad}}}} = 0\, {\text{No interaction}}} \hfill \\ {{\text{E}}_{{{\text{ad}}}} < 0\, {\text{Attraction}},{\text{ bonding}}} \hfill \\ \end{array} } \right.$$

**Table 5:** System energy and adsorption energy of BN under different adsorption positions on the surface of Al–Al_2_Cu (0 0 1).

Adsorption location	The system can be adsorbed after adsorption $\text E_{\text{BN}/\text{Al}_2\text {Cu} \,\left( {0 \, 0{ 1}} \right)}$/eV	Adsorption substrate separate system energy $\text E_{\text{Al}_2\text {Cu} \,\left( {0 \, 0{ 1}} \right)}$/eV	Adsorbate individual system energy E_BN_/eV	Adsorption energy E_ad_/eV
B-Bridge	−8198.01	−7830.52	−360.91	−6.58
B-Top	−8197.84			−6.41
B-V_1_	−8195.89			−4.46
B-V_2_	−8195.72			−4.29
N-Bridge	−8198.07			−6.64
N-Top	−8195.76			−4.33
N-V_1_	−9198.02			−6.59
N-V_2_	−8197.99			−6.56

**Table 6:** System energy and adsorption energy of BN under different adsorption positions on Cu-Al_2_Cu (0 0 1) surface.

Adsorption location	The system can be adsorbed after adsorption $\text E_{\text{BN}/\text{Al}_2\text {Cu} \,\left( {0 \, 0{ 1}} \right)}$/eV	Adsorption substrate separate system energy $\text E_{\text{Al}_2\text {Cu} \,\left( {0 \, 0{ 1}} \right)}$/eV	Adsorbate individual system energy E_BN_/eV	Adsorption energy E_ad_/eV
B-Bridge	−9657.06	−9288.21	−360.91	−7.94
B-Top	−9652.09			−2.97
B-V	−9653.83			−4.71
N-Bridge	−9656.68			−7.56
N-Top	−9652.51			−3.39
N-V	−9656.16			−7.04

**Figure 14:**
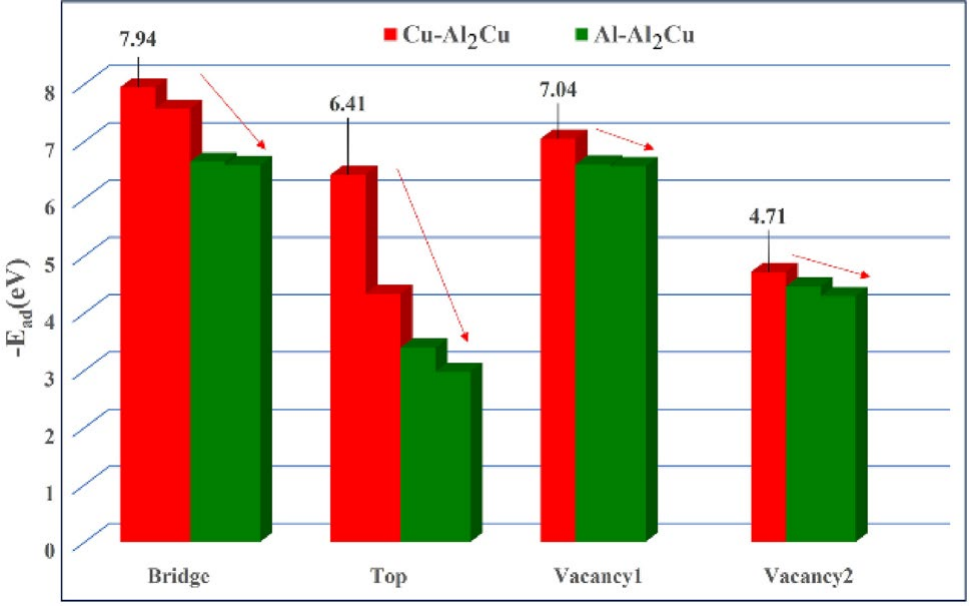
Adsorption energies of BN at the same adsorption sites on the Al_2_Cu (0 0 1) surface.

**Figure 15:**
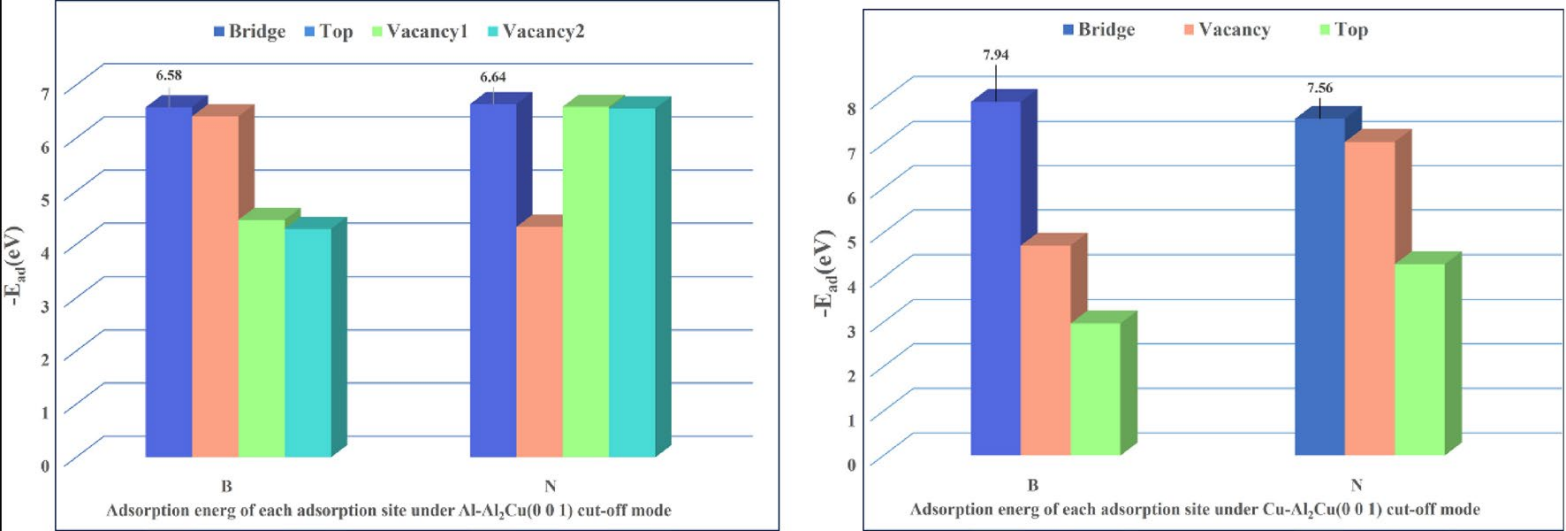
Adsorption energy of BN at different adsorption sites on Al_2_Cu (0 0 1) surface.

Observing Figs. 14 and 15, it can be found that for the same adsorption position, BN is easy to be adsorbed on the surface of Cu–Al_2_Cu (0 0 1) at the bridge position (B) and the vacancy position (V), and BN is more likely to be adsorbed on the surface of Al–Al_2_Cu (0 0 1) at the top position. Under different adsorption positions, BN has the lowest adsorption energy (highest absolute value) system structure at the bridge position (B) is the most stable, and it is easier to be adsorbed on the Cu–Al_2_Cu (0 0 1) surface. Moreover, on the Al–Al_2_Cu cut-off surface, the N atoms in the adsorbed configuration tend to bind to the Al atoms, and on the Cu–Al_2_Cu cut-off surface, the B atoms in the adsorbed configuration tend to bind to Cu atoms. This indicates that the adsorption behavior of BN affects the stability of the alloy system after its incorporation, while the bonding between atoms under adsorption facilitates the formation of stable structures within the alloy. Since the adsorption capacity of an adsorbate molecule is related to the chemical bonds formed between atoms on the surface of the alloy, the electronic structure of the adsorbed configuration can be further calculated to obtain the bonding.

#### Band structure and State density

**Figure 16:**
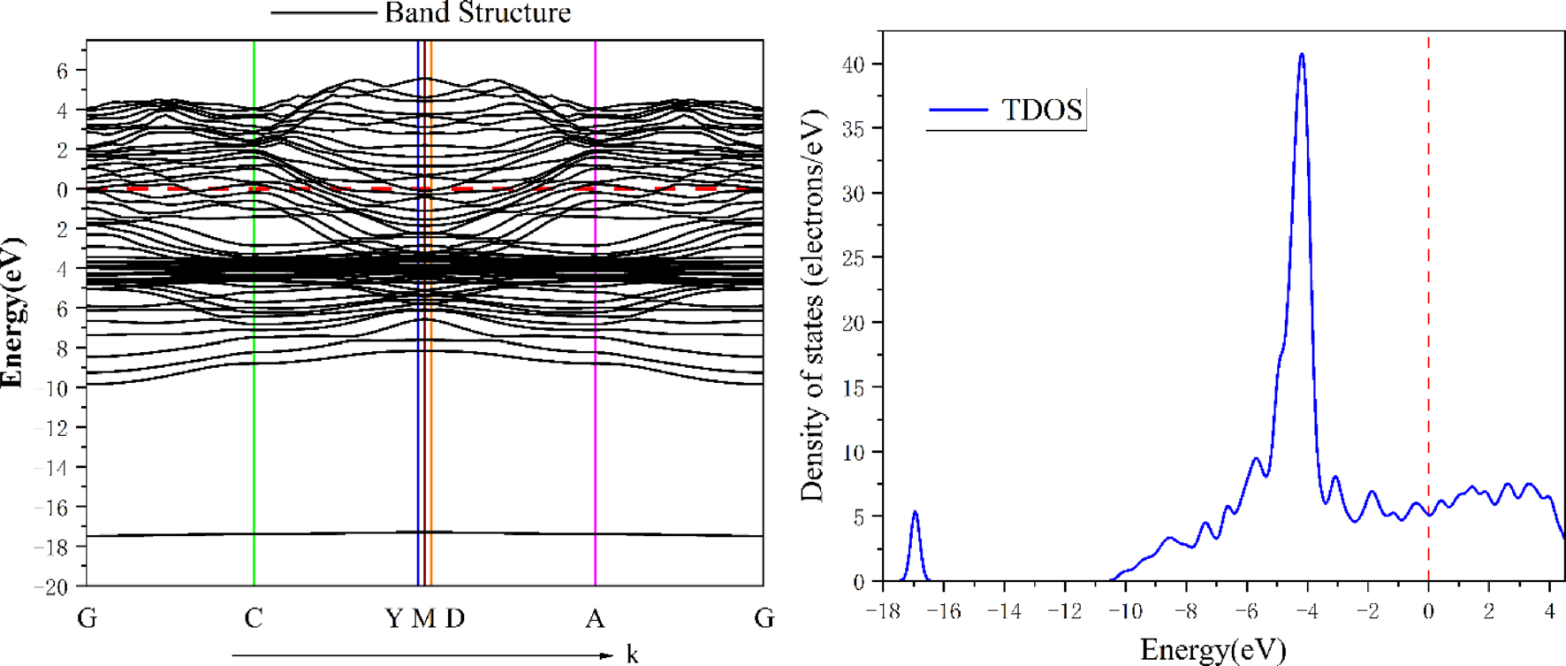
Energy band structure and total density of states of BN adsorbed on Al_2_Cu(0 0 1) surface.

**Figure 17:**
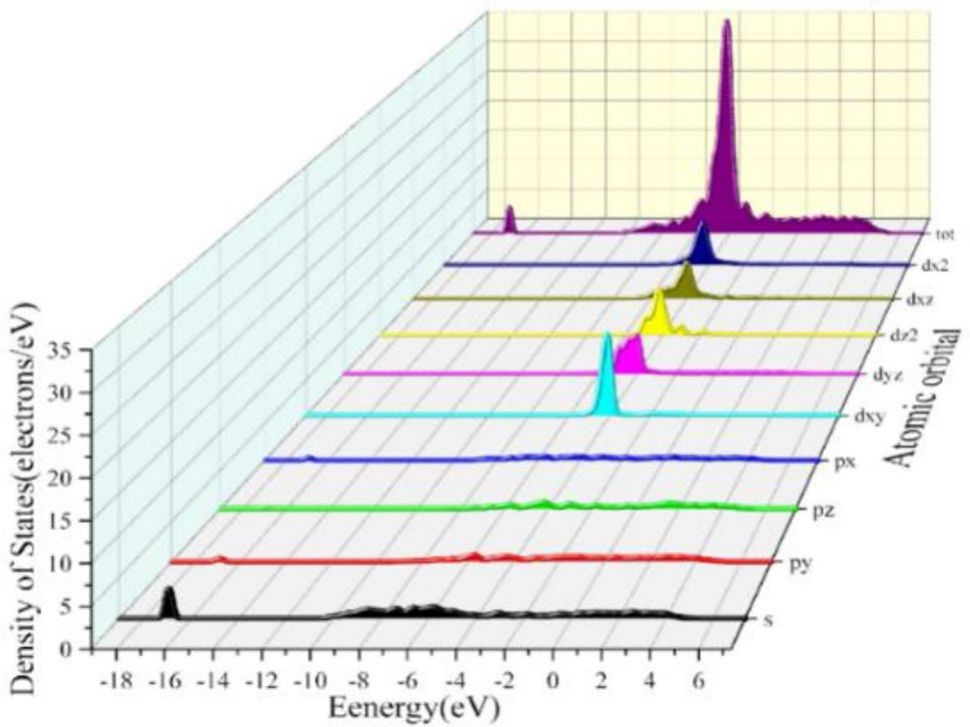
Total partial wave density of states (PDOS) of the system after adsorption.

**Figure 18:**
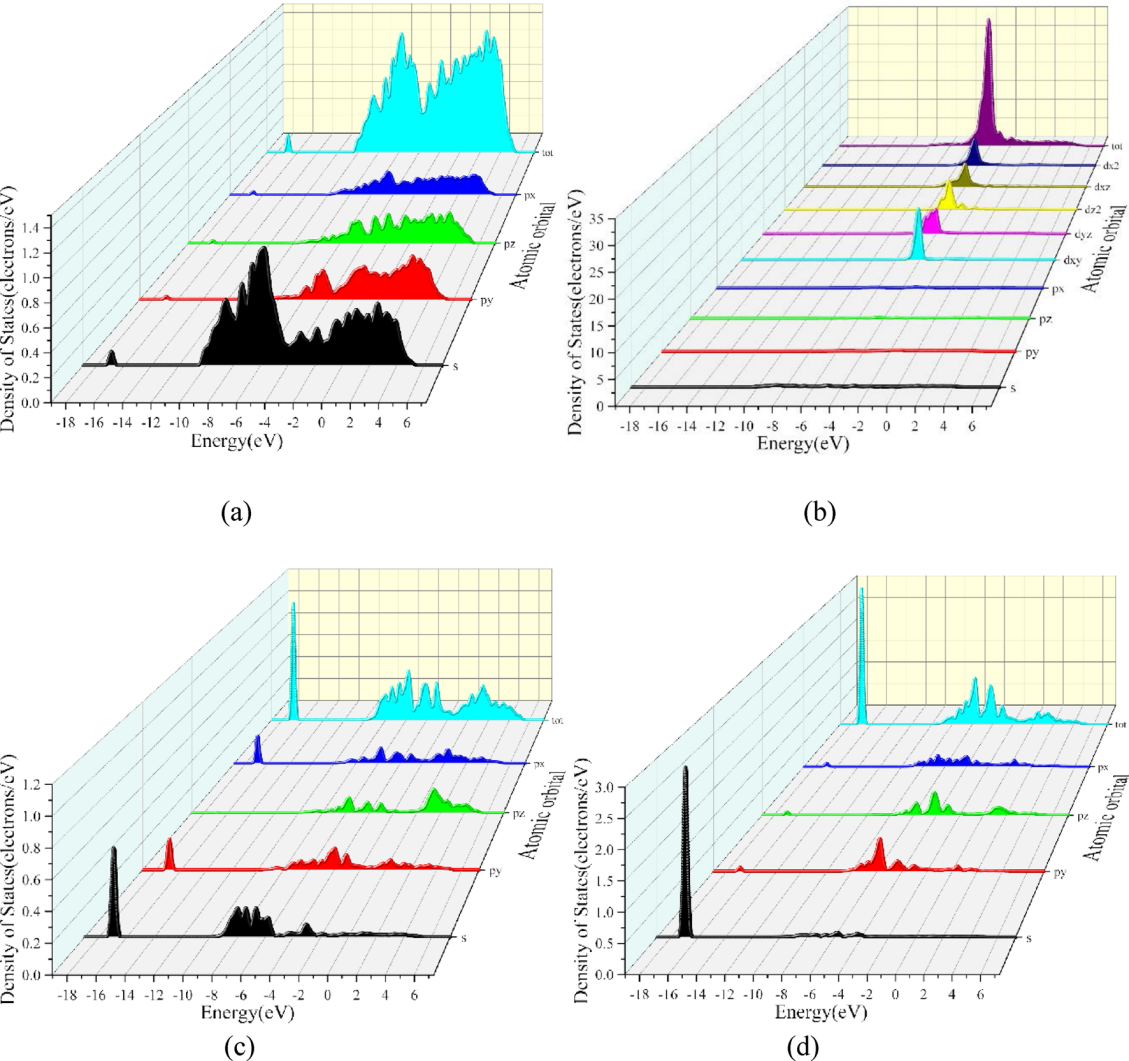
Projection of BN on the fractional wave state densities and atomic orbitals of individual atoms adsorbed on the Al_2_Cu (0 0 1) surface: (**a**) PDOS-Al; (**b**) PDOS-Cu; (**c**) PDOS-B; (**d**) PDOS-N.

In order to better understand the electronic structure and properties of the Al/Cu–Al_2_Cu (0 0 1) system after BN adsorption, the energy band structure, the density of states, and the projections onto each atomic orbital were calculated as shown in Figs. 16, 17, and 18, where the Fermi energy level (E_f_ = 0 eV) is indicated by the red dashed line. As shown in Fig. 16, in the total density of states (TDOS) showing the specific composition of the electronic states in the energy band structure, the peak with the smallest valence band energy occurs at − 16.94 eV, while the peak with the densest energy state occurs at − 4.18 eV. A "pseudo-energy gap" can also be found in the DOS diagram, i.e., the DOS values between the left and right spikes at the Fermi energy level are also not zero, reflecting the covalent bonding state of the alloy system^61^. To further understand the bonding within the alloy and the contribution of electrons between the atomic orbitals, the total partial wave density of states (PDOS) of the system after adsorption and the projections of the partial wave densities of the individual atoms in the system onto the atomic orbitals were calculated as shown in Figs. 17 and 18.

From the total partial wave density of states (PDOS) of the system in Fig. 17, it can be seen that the free electrons in the alloy are mainly composed of d-orbital electrons as well as a tiny number of *s*- and *p*-orbital electrons. To explain the stability of macroscopic objects, it is necessary to take into account the contribution of free electrons in certain fixed atomic orbitals. Based on the arrangement of electrons in the valence layer outside the nucleus of each atom and in conjunction with Fig. 18-b, it is found that the electrons in the *d* orbitals of the system come from the electronic contribution of the 3*d* state of the Cu atom and are most abundant at − 4.18 eV. Here the density of states forms a sharp peak, and the corresponding band is narrow, indicating the strong nature of electron localization in the 3*d* orbitals. From Fig. 18-a,-c,-d, the electrons in the *s* and *p* orbitals are dominated by valence electrons from Al, B, and N atoms. Where electrons in the *s* orbitals of the conduction band are exclusively contributed by Al-3*s*, electrons in the *p* orbitals are contributed by Al-3*p*, B-2*p* and to a lesser extent N-2*p*. The electrons in the *s* and *p* orbitals in the valence band part are contributed by each atom, while at the energy level − 4.18 eV they are contributed by the Al-3*s* and 3*p* and Cu-3*d* and 4*s* states of the system with more atoms, and the B and N atoms contribute relatively little. However, at the lower energy level of − 16.94 eV, the electrons in the *s* orbitals are mainly contributed by the N-2*s* state, and the electrons in the *p* orbitals are mainly contributed by the B-2*p* state, which is the reason why the energy band structure in Fig. 16 will form an isolated energy band here. It is inferred that there is a strong orbital hybridization between the B-2*p* orbitals, N-2*s* orbitals and other atoms throughout the low-energy state range, and the bonding of the system can also be seen here, which is not only the existence of strong Al–Cu covalent bonding in the structure, in which there are additional strong covalent bonding between the B and N atoms and the Al and Cu atoms. In view of the differences in bonding between the atoms within the different adsorption configurations of the alloys, it is possible to analyze them in-depth by means of the differential charge density, i.e., the redistribution of charge after the atoms have formed a system (cluster).

#### Differential charge density

**Figure 19:**
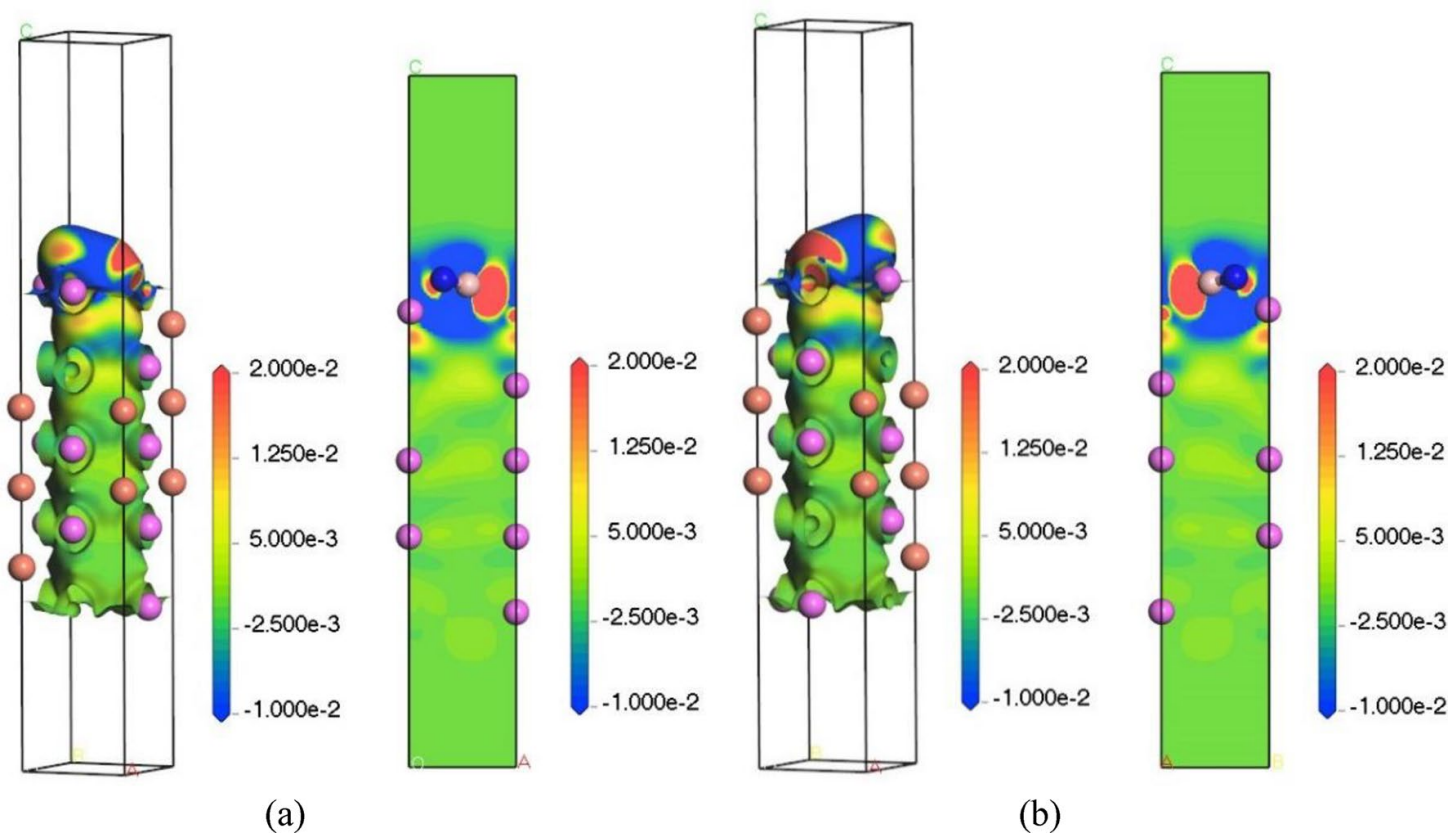
Differential charge density adsorbed by BN on the surface of Al–Al_2_Cu (0 0 1): (**a**) B-Bridge; (**b**) N-Bridge.

**Figure 20:**
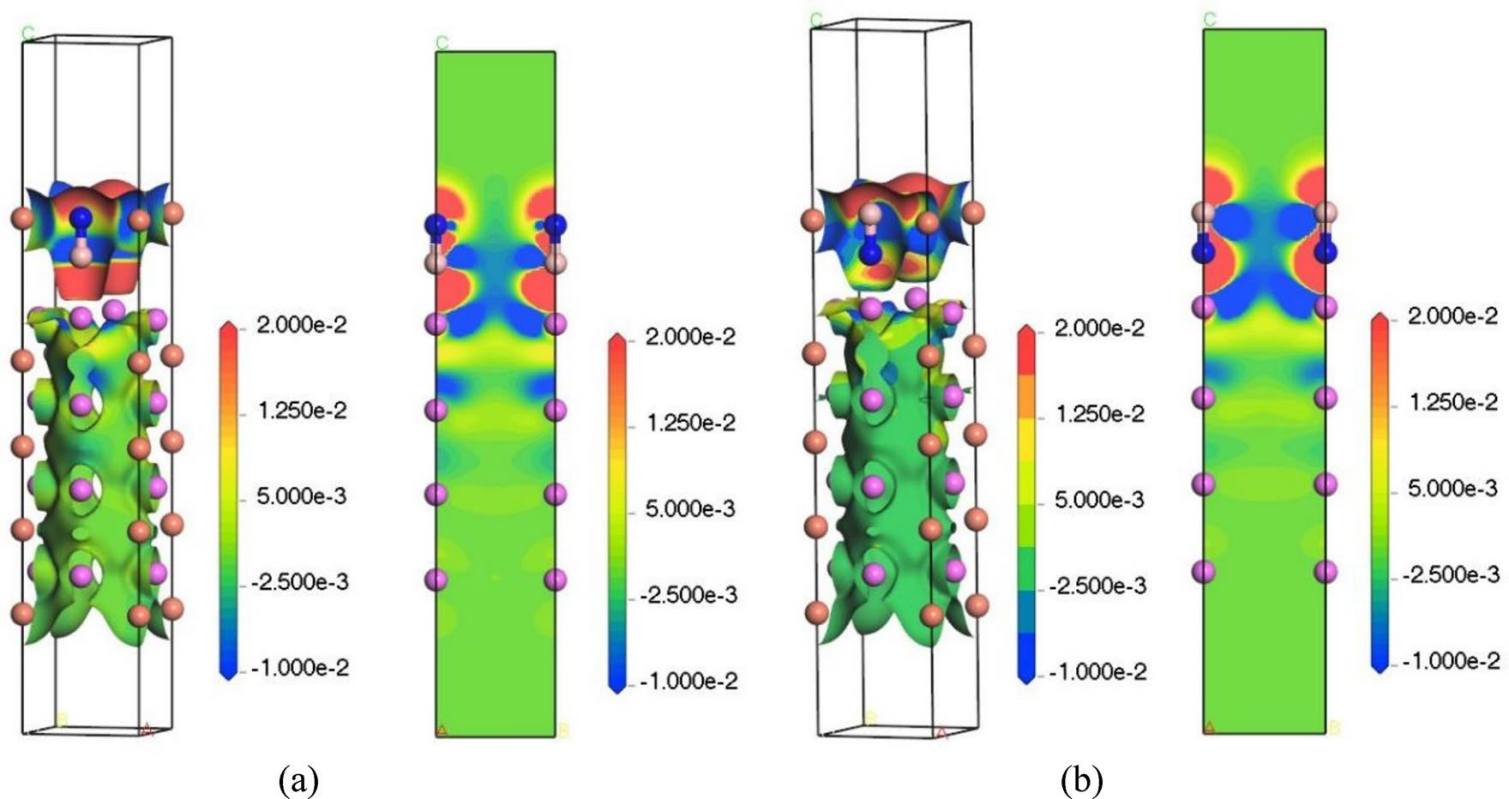
Differential charge density adsorbed by BN on the surface of Cu–Al_2_Cu (0 0 1): (**a**) B-Bridge; (**b**) N-Bridge.

In order to understand the bonding between the atoms in the BN and Al_2_Cu (0 0 1) surfaces more intuitively and graphically, as well as the strength of the bonding polarity, the four bridge adsorption configurations with the lowest adsorption energies in the model were selected, and their differential charge densities were calculated as shown in Figs. 19, 20. The right figs showed slices of the differential charge density map, where the specific spatial distribution of charge aggregation and loss can be visualized with different colors^62^. In the figs, the red area indicates that the electron is obtained when the electron density increases, and the blue area indicates that the electron density decreases and the electron is lost, and it can be clearly seen that there are electrons transferred from Al atoms and Cu atoms to BN molecules on the surface of Al_2_Cu (0 0 1). It can be judged that the adsorption method is chemical adsorption, that is, the adsorbent reacts chemically with the surface of the adsorbed substrate to form a strong adsorption chemical bond, such as Al–B bond and Cu–N bond.

Secondly, it is found that in the surface adsorption model of Al–Al_2_Cu (0 0 1), the charge density between Al–B is high and the charge density between Al–N is low, so it is easier to form bonds between Al–B, while the role between Al–N is not obvious. In the Cu–Al_2_Cu (0 0 1) surface adsorption model, it is found that the charge density between Cu–N is high, and the charge density between Cu–B is reduced, so it is easier to form bonds between Cu–N. The formation of Al–B bond and Cu–N bond indicates that there is a strong interaction between BN and Al_2_Cu (0 0 1) surface, which is conducive to the adsorption of BN on the alloy surface.

Furthermore, Al and B are both common group elements, and binding to the main group elements usually forms covalent compounds. For example, AlB_2_ can be formed between Al and B^63^. B atoms have a larger electronegativity than Al atoms, attracting more electrons in the compound AlB_2_, which is also responsible for the large overlap of the Al and B charge densities in Fig. 19. At the same time, the electron clouds of Cu and N are greatly overlapped and they jointly occupy some sites to form chemical bonds, indicating that there is also a strong interaction between Cu–N. Thus, after the addition of boron nitride ceramic particles to the Al–Copper alloy, because the B and N atoms in BN are themselves higher electronegative, and at the same time, the interaction between Al and Cu is enhanced to form a stronger covalent bond, making the architecture more stable, which is in agreement with previous calculations.

## Discussion

### The mechanism of action of BN on the tensile strength of alloys

**Figure 21:**
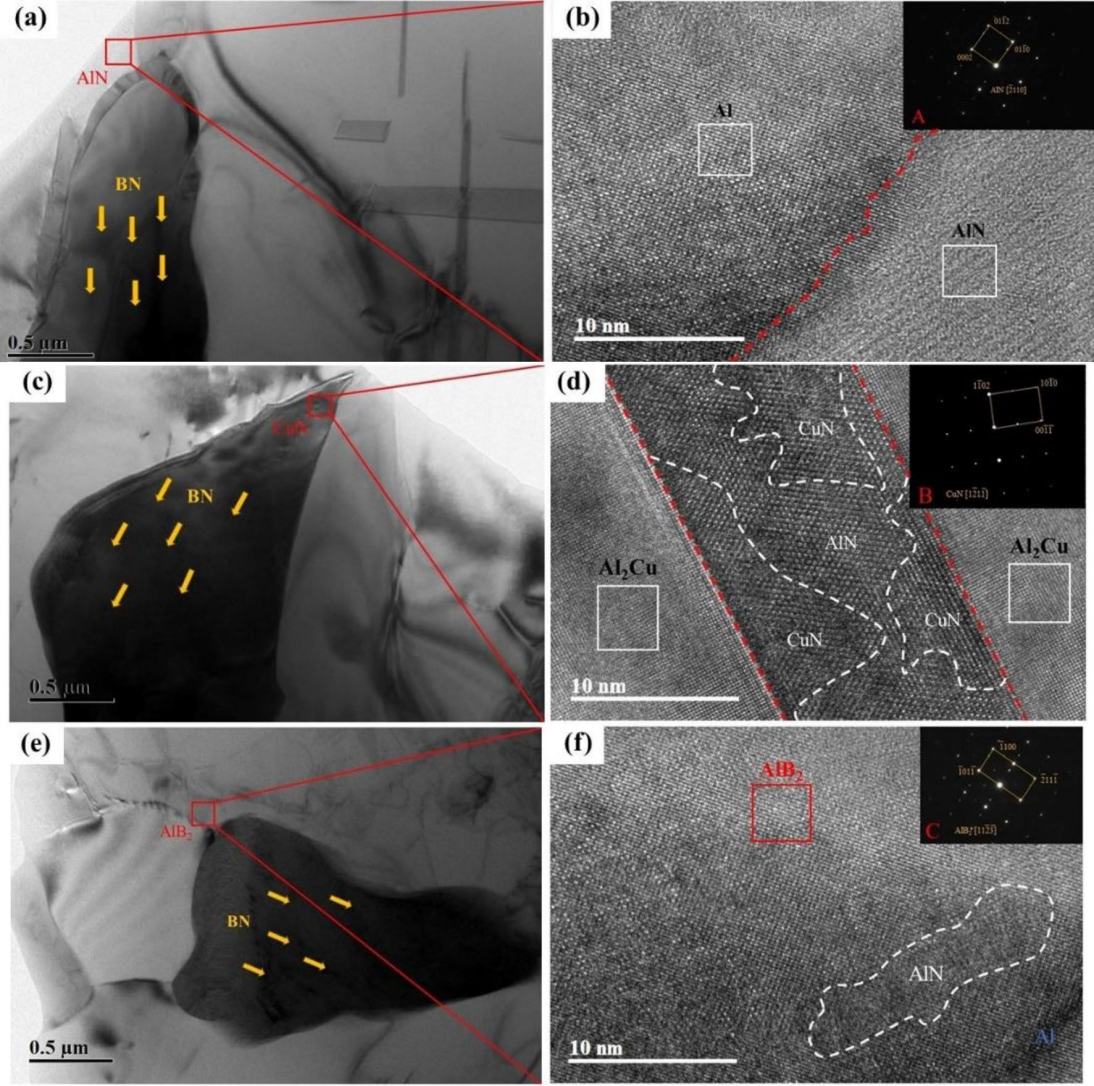
HRTEM micrographs of Al–5Cu–Mn–0.5BN alloys: (**a**), (**c**), (**e**) enhanced phases AlN, CuN & AlB_2_ on the matrix; (**b**), (**d**), (**f**) structures of the enhanced phases in high-resolution Fourier Transform (FFT) mode, insets show electron diffraction patterns.

**Figure 22:**
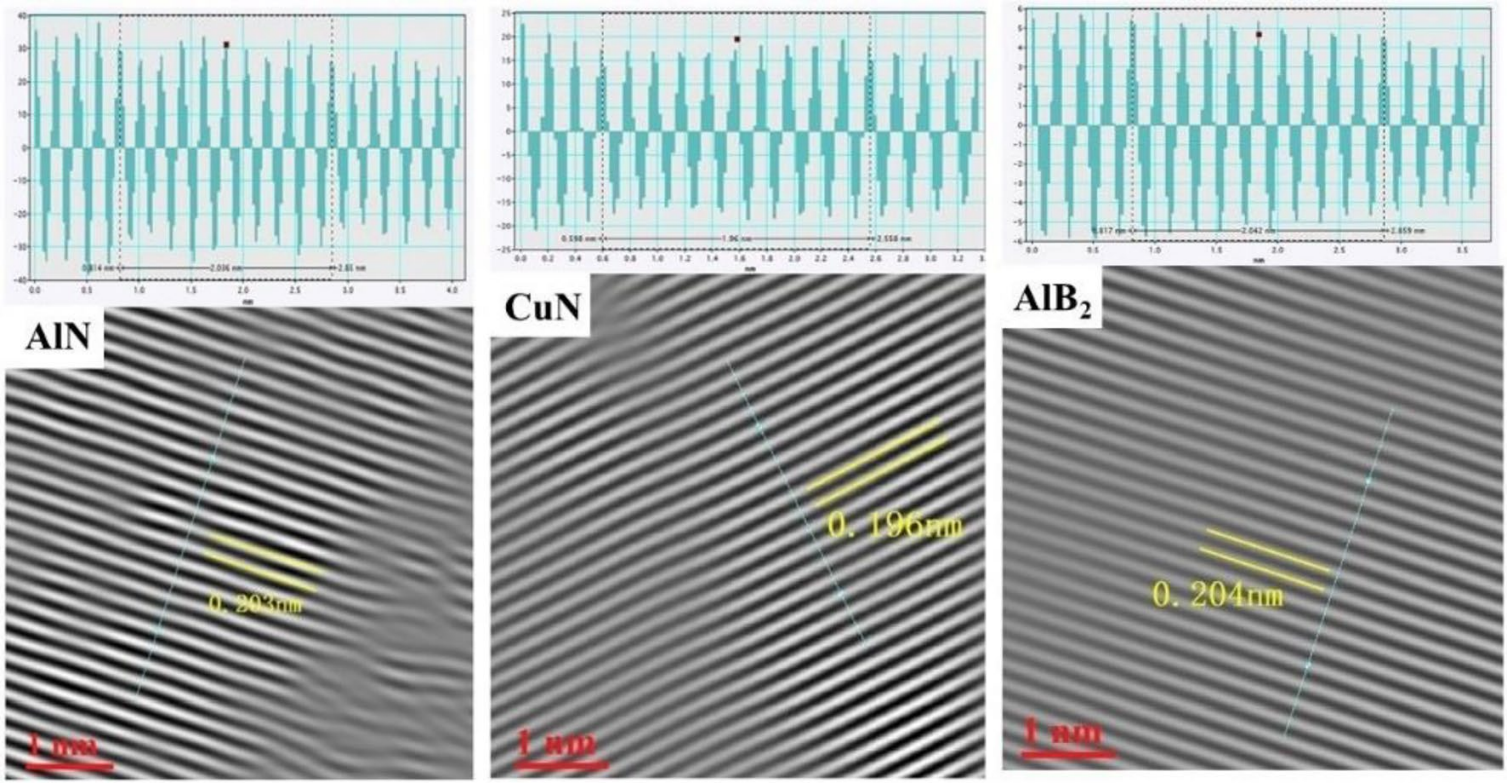
High resolution Inverse Fourier Transform (IFFT) and crystal surface calibration of the enhanced phase.

The influence of boron nitride particles on the electronic structure of the alloy is understood by means of simulation calculations. The addition of boron nitride particles leads to the formation of localized electronic states in the alloy, changing the Fermi level and the electron density of states in the second phase of the original alloy Al_2_Cu, whose stability is closely related to the distribution of the electron density of states at the Fermi level. In the simulation calculation results, after adding boron nitride particles, the total electron state density of the metallized system does not appear a singular spike at the Fermi level, and the value of the state density is relatively flat at the Fermi level, at this time, the extranuclear electrons have local properties and are not bound, can move freely near the local potential field of Al and Cu atoms, the stability of the system is good, and the phase transition will not occur easily. This can also be seen from the differential charge density plots, where the valence electrons of the atoms in the original alloy are transferred to the BN in the adsorption model with different cutoff modes, forming stable covalent alloys such as AlB_2_, AlN, and CuN. These are the high-intensity phases in the matrix, and their strong bonding plays an essential role in enhancing the strength of the composite. To observe the microstructure of these compounds, samples 2–5 were photographed by transmission electron microscopy (TEM), and the results are shown in Figs. 21 and 22.

Figure 21 showed the evolution of the BN/Al interface from TEM morphology to FFT images, and it is observed that three interfacial products are formed at the interface, as shown in Figs. b, d, and f (AlN, CuN, and AlB_2_). Based on the interatomic charge transfer in the simulation calculations (Figs. 19, 20), it is hypothesized that the crystal layer contains abundant nitrogen and boron sources to provide reactions for the interface. In addition, the "adsorption" between BN and Al_2_Cu plays a crucial role in the bonding interface between them, and the surrounding parts of the BN/Al interface in the HRTEM images are clean and tightly bonded, and the corresponding electron diffraction patterns (insets A, B, and C) show the detailed crystalline structures of the compounds. The AlN–Al interface orientation relationship can be expressed as [110]_Al_//[2110]_AlN_, [111]_Al_//[0112]_AlN_; the CuN–Al interface orientation relationship can be expressed as [110]_Al_//[1211]_CuN_, [111]_Al_//[1010]_CuN_; The AlB_2_–Al interface orientation relationship can be expressed as: [110]_Al_//[1123]_AlB2_, [111]_Al_//[2111]_AlB2_. The images shown in insets A, B, and C indicate that the interface is parallel coherent, suggesting a better match between the BN/Al_2_Cu interfaces, which effectively increases the adsorption energy, and this is also consistent with the theoretical calculations of the adsorption energy. Figure 22 shows the planar spacing of the reinforcing phases AlN, Cu, and AlB_2_ measured from the IFFT images, which are 0.203 nm, 0.196 nm, and 0.204 nm, respectively, and the edge dislocations formed by the reinforcing phases at the Al-based interfaces, whose distribution along the grain boundaries enhances the strength properties of the material, are observed from the enlarged IFFT images.

**Figure 23:**
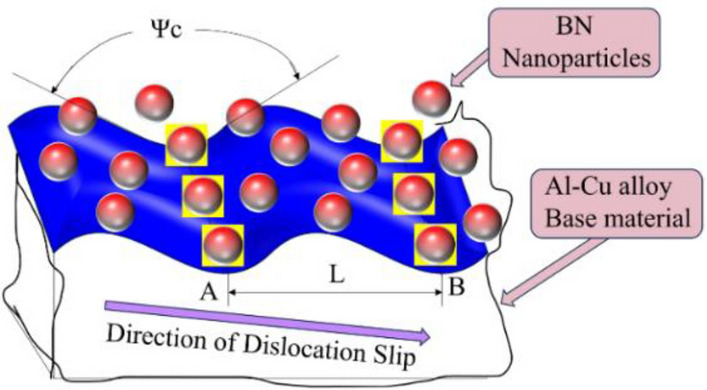
"Pinned" reinforcement effect.

Secondly, after the addition of BN, edge dislocations were formed around the Al_2_Cu(BN) particles. which is a similar "pinned" effect of boron nitride particles as a reinforced phase in aluminium alloys^64^. When the stress is loaded into the alloy, the boron nitride particles are able to resist crystal slip, increasing the density and kinematic resistance to dislocations and thus increasing the yield and tensile strength of the alloy. The mechanism model is shown in Fig. 23, and the dislocation generated in the slip direction is tied when it hits two nail points A and B (BN particles) with a spacing of L. When external stress occurs, the dislocation section between A and B will bend, and as the stress increases, the bending of the dislocation section becomes more serious. Due to the driving action, the dislocation curve angle *Ψ*_*C*_ on both sides of the nail point B is also reduced. Then at this time, if the dislocation crosses the nail point, it needs a greater driving force to return to its original state and continue to move forward, so that the yield strength and tensile strength of the alloy material are improved, which is also the strengthening effect of the nail point.

In addition, it has been shown that the chemical reaction at the interface between the reinforcing phase and the metal matrix can significantly increase the interface strength^65^. Strong interfaces also exist between the Al matrix and the reinforcing phase (BN particles) when new compounds are formed at the Al_2_Cu phase interface, and the specific orientation relationship produces high adsorption energies and strong interfacial bonds. For example, the formation of an amorphous transition layer between BN and Al matrix is a strong interfacial bonding^66^. In addition, an important condition for the high bond strength of the compound is the large adsorption energy between the two phases. In this study, compared to other adsorption positions, the system energy of BN adsorbed at the bridge position is greater between the large adsorption energy, as shown in the results of simulation calculations of the system energy of each adsorption configuration (Fig. 15), and the strength of the composite material is thus enhanced.

### Mechanism of BN action on surface hardness of alloys

**Figure 24:**
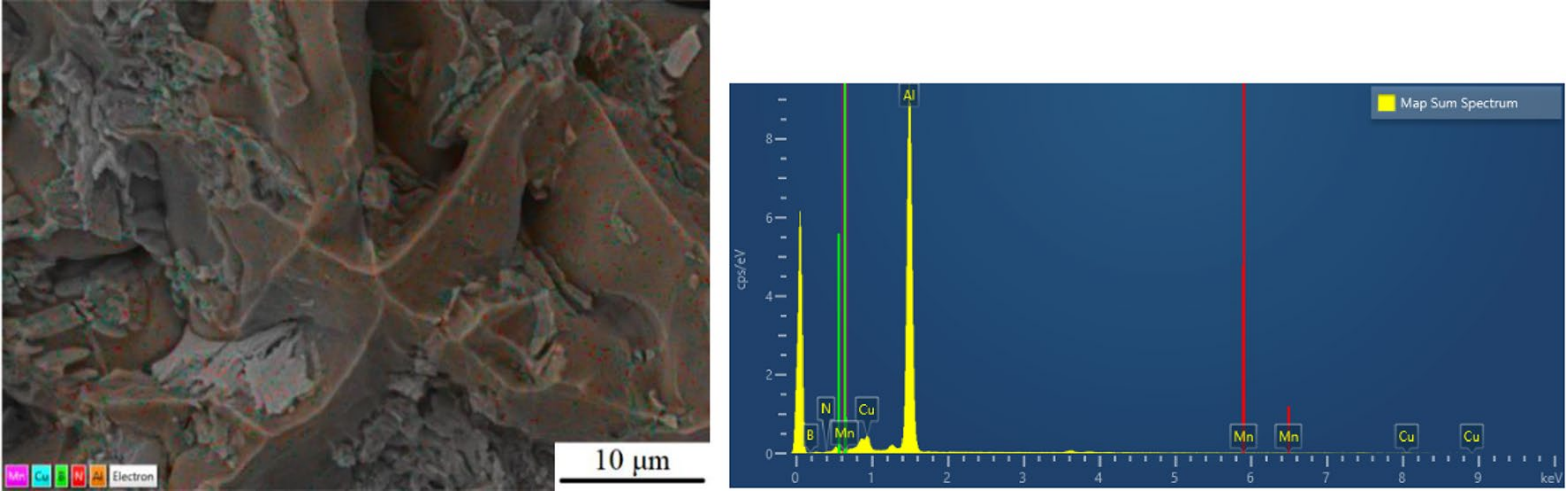
Specimen 2–2 EDS and Energy spectra.

Although the simulation results showed that the B and N atoms enhance the interaction between Al and Cu, forming a stronger covalent bond. But, in casting experiments, since the temperature of about 750 °C is not enough to dissolve the BN, the distribution of BN particles in the calculation model may be different from the bonding, so it is necessary to characterize the BN by transmission and energy spectroscopy. The fracture morphology of specimen 2–2 with optimum hardness after addition of BN particles was scanned by X-ray energy spectrometry analysis (EDS), and its elemental distribution and energy spectrum are shown in Fig. 24. It can be seen that in this specimen the B and N elements are uniformly distributed over the break in the matrix. The reason for this is that the molten aluminium in the casting process makes it easier to introduce BN particles into the alloy and mix them better with the matrix. At the same time, excellent homogeneity is provided due to the lubricating nature of the BN particles, and stirring prevents agglomeration of the particles in the melt, leading to better dispersions and improved hardness of the alloy material.

**Figure 25:**
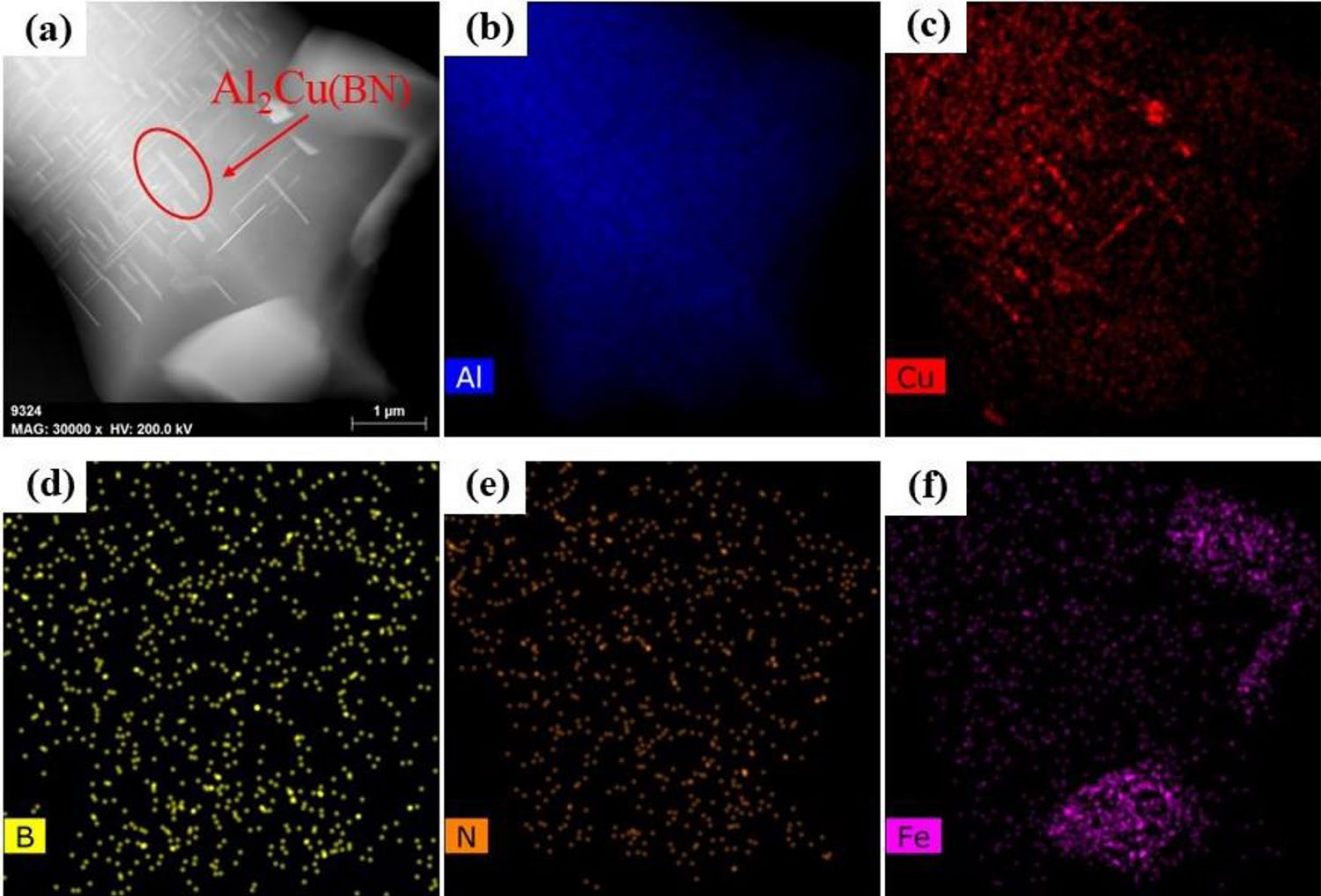
Mapping of Al_2_Cu (BN) particles in the dislocation region to the elements.

​According to the SEM analysis in the tensile test, this specimen exhibits a quasi-dissociative fracture, with torn ribs and lobes on the fracture surface. In the X-ray spectroscopy (EDS) analysis, B and N elements were found to be contained and widely distributed on the tearing prongs by surface scanning, and comparing the toughness fracture of the specimens without added BN particles (as shown in Figs. 8, 9), it was hypothesized that the appearance of tearing prongs and quasi-dissociative fracture were related to the added BN particles, and that the fracture of the specimens had brittleness and had an effect on the hardness. The dispersion of BN particles helps to dissolve the B and N elements in the aluminium-copper alloy, changing the grain structure and grain boundary distribution, refining the grain size (as shown in Fig. 12,-a1,-a2), and forming the reinforced phase grains, such as AlB_2_, AlN and CuN, inside the composite material, resulting in a more homogeneous organization at the grain boundaries, and the hardness of the material has been significantly improved. To further clarify the distribution of elements around the Al_2_Cu particles, the microstructure in this sample was observed by TEM and the results are shown in Fig. 25. In the figure, B and N elements are uniformly distributed in the matrix, and the N elements enriched around the Al_2_Cu(BN) particles are less than the B elements, suggesting that N only occupies the vacancies of Al_2_Cu and does not change the physical phase of Al_2_Cu. This is consistent with the adsorption energy calculations (Fig. 15), where the absolute values of the adsorption energies of the vacancies in the N-Al_2_Cu (0 0 1) system are higher than those of the B-Al_2_Cu (0 0 1) results. Secondly, the dislocation in the sample was characterized and it was found that dislocation strips were formed at the aggregation of Al_2_Cu(BN) particles, indicating that the incorporation of BN would increase the dislocation density in Al_2_Cu and strengthen the hardness of the material.

In addition, boron nitride is a tough ceramic particle, and the hard phase content in the alloy is enhanced by its addition^67^. The presence of hard phases hinders lattice slip and dislocation motion, leading to increased complexity of grain boundaries. The fine size of the boron nitride particles, however, facilitates the formation of a diffuse hard phase in the aluminium alloy matrix, thereby increasing the hardness of the material. Also, at the atomic level, hexagonal boron nitride (hBN) adopts a honeycomb structure *sp*^2^ covalent bonding^68^, which is attributed to the phenomenon of partial overlap of the fractional wave state densities of the B and N atoms in the valence band, as well as at the lower energy levels (shown in Fig. 18), with hybridization of the electrons in the *s* and *p* orbitals. This is extremely similar to the graphene hexagonal structure^69^, where both atoms in BN form three bonds and are attached to neighboring atoms. This structure makes the B-N bond particularly strong and extremely difficult to break. When BN particles are dissolved into the matrix crystal, they occupy vacancies in the lattice as well as taking the place of the rest of the atoms in the alloy, introducing additional lattice distortions and a localized stress field increasing the resistance to motion of the slip and further enhancing the hardness of the material.

### Mechanism of BN action on elongation of alloys

In the experimental results, it was found that the elongation of the specimen was reduced by the addition of the reinforcement BN particles. This is due to the fact that the introduction of boron nitride particles leads to a certain degree of lattice distortion, which may hinder the motion of the dislocations from sliding as freely as in the absence of particles. When the material is subjected to a force, the particle becomes a stress concentration point, resulting in localized stress accumulation and dislocations that cannot slip and are difficult to deform plastically, thus reducing elongation. In addition, the presence of boron nitride particles as a reinforcing phase, which commonly has high hardness and stiffness^70^, makes it more difficult for the alloy material to ductile under stress. As a result, the overall recombination plastically is suppressed.

**Figure 26:**
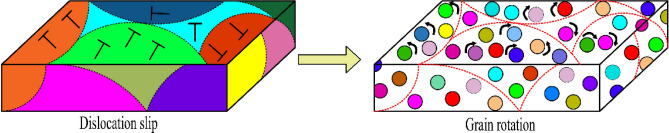
Plastically mechanism.

Although numerous movable dislocations in the alloy are hindered, due to the tiny size of the BN particles, the source of dislocations attaching to the BN particles is usually not at grain boundaries but located inside the grains, and the mechanism of deformation changes from dislocation slippage to grain rotation as shown in Fig. 26. When these dislocation sources are effectively plugged inside the grain, the grain boundary area increases and zigzagging occurs, so that the stress concentration is smaller, plastic deformation can be uniformly dispersed in further grain interior, preventing crack extension, both to ensure the strength of the alloy material without causing a significant decline in elongation properties. Meanwhile, according to the calculation results of adsorption energy (Fig. 14), it is shown that after the incorporation of BN particles in Al–5Cu–Mn–0.5BN, stable adsorption of BN occurs on the surface of Al_2_Cu (0 0 1), and the higher absolute value of adsorption energy makes the interaction between atoms in BN particles and the solid surface strong, and BN particles maintain a stable position with the substrate and inhibit the offset of cutoff surface, which helps to maintain the elongation properties of the alloy. In addition, the formation of the Al_2_Cu phase in the original alloy can effectively enhance the load transfer of BN in aluminium-based metal composites because the "anchoring" effect prevents local interfacial slip^71^, The hexagonal boron nitride (hBN), which has a layered structure, is unaffected by mobility in the lattice, preventing corrosion and loosening of the grain boundaries, which additionally ensures the elongation of the material.

## Conclusion

In this study, Al–5Cu–Mn–0.5BN alloy was studied through casting experiments to compare and analyze the effect of boron nitride (BN) particles on the properties of cast aluminium-copper alloy materials. To elaborate the enhancement mechanism of BN ceramic particles in cast aluminium-copper alloys, a first-principles approach was used to establish a computational model and analyze the adsorption of BN on the surface of the second phase, Al_2_Cu (0 0 1), as well as the nature of the electronic structure after adsorption. The combined experimental and computational results lead to the following conclusions:

1. From the experimental results, the strength and hardness of the composites were significantly enhanced by the addition of BN particles, by 8.5% and 10.2%, respectively, while the plastically of the composites was reduced. Likewise, the addition and uniform distribution of BN reduces the particle size in the alloy. This facilitates grain refinement.
2. Calculated results showed that the adsorption positions of BN on the Al_2_Cu (0 0 1) surface are all capable of stable chemical adsorption, and the adsorption reaction can proceed autonomously. Among the different cutoffs, the adsorption energy occurring on the Cu–Al_2_Cu (0 0 1) surface is smaller and the adsorption structure is relatively more stable. For different adsorption sites, BN has the smallest adsorption energy of –7.94 eV at the bridge site and is the most easily formed. In terms of the electronic structure, in the low energy regime, the charge is transferred to the B and N atoms due to the electronegativity, and there are strong covalent bonds within the alloy.
3. From the enhancement mechanism, the addition of ceramic particles BN can change the crystal structure of the alloy and the distribution of grain boundaries, the formation of the corresponding AlB_2_, AlN, and CuN enhancement phase in the matrix. These enhancement phases in the casting of aluminium-copper alloys can play a role in resisting the crystal slippage, increase the role of dislocation density and resistance to movement, which increases the tensile strength and hardness of the alloy. For plastically, there are also a number of counteracting factors such as dislocation source plug accumulation, solid surface inhibition, and other factors such that the plastically reduction is small.

### Supplementary Information

Supplementary Information.

## Data Availability

The data that support the findings of this study are available from the corresponding author upon reasonable request.
