# Supplementary material for: Influence of nano-BN inclusion and mechanism involved on aluminium-copper alloy
Source: Sci Rep. 2024 Mar 16;14:6372. doi: 10.1038/s41598-024-56986-3 (PMC10944483; doi:10.1038/s41598-024-56986-3)
Supplement: Supplementary file 1 — Supplementary Information. [file 41598_2024_56986_MOESM1_ESM.pdf]

In this study, a total of 14 adsorption configurations of BN adsorption Al_2Cu (0 0 1) surfaces were established, and the following are the details of the input files INCAR, POSCAR, KPOINTS, and POTCAR performed by the VASP software package, among which the POSCAR files are shown as the four unit cell models with the largest adsorption energy values.

INCAR:

SYSTEM=ZL201_BN
ISTART = 0
ENCUT = 600
IBRION = 2
EDIFF = 1E-5
EDIFFG = -0.01
NSW =140
ISIF = 3
PREC=Accurate
ALGO=Normal
IALGO=38
POTIM = 0.1
ISMEAR = 0
SIGMA = 0.1

KPOINTS:

KPT-Resolved Value to Generate K-Mesh: 0.020
0
Gamma
10 10 1
0.0 0.0 0.0

POTCAR: cat ~bin/paw_pbe/Al/POTCAR ~bin/paw_pbe/Cu/POTCAR ~bin/paw_pbe/B/POTCAR ~bin/paw_pbe/N/POTCAR > POTCAR

POSCAR 1:

Al_2Cu(0\0\1)-B-bridge

1.0

4.1051998138 0.0000000000 0.0000000000

0.0000000000 4.1051998138 0.0000000000

0.0000000000 0.0000000000 26.4566001892

Al Cu B N

10 4 1 1

Selective Dynamics

Direct

-0.000000000 0.500000000 0.224730006 T T T

0.000000000 0.500000000 0.334019988 T T T

-0.000000000 0.500000000 0.443300020 F F F

0.000000000 0.500000000 0.552590002 T T T

-0.000000000 0.500000000 0.661869998 T T T

0.500000000 -0.000000000 0.224730006 T T T

0.500000000 0.000000000 0.334019988 T T T

0.500000000 -0.000000000 0.443300020 F F F

0.500000000 0.000000000 0.552590002 T T T

0.500000000 -0.000000000 0.661869998 T T T

0.000000000 0.000000000 0.279379990 T T T

-0.000000000 -0.000000000 0.388660004 F F F

0.000000000 0.000000000 0.497949986 F F F

0.000000000 0.000000000 0.607229982 T T T

0.250000000 0.250000000 0.735559998 T T T

0.250000000 0.250000000 0.783900053 T T T

POSCAR 2:

Al_2Cu(0\0\1)-N-bridge

1.0

4.1051998138 0.0000000000 0.0000000000

0.0000000000 4.1051998138 0.0000000000

0.0000000000 0.0000000000 26.4566001892

Al Cu N B

10 4 1 1

Direct

-0.000000000 0.500000000 0.224730006 T T T

0.000000000 0.500000000 0.334019988 T T T

-0.000000000 0.500000000 0.443300020 F F F

0.000000000	0.500000000	0.552590002 T T T
-0.000000000	0.500000000	0.661869998 T T T
0.500000000	-0.000000000	0.224730006 T T T
0.500000000	0.000000000	0.334019988 T T T
0.500000000	-0.000000000	0.443300020 F F F
0.500000000	0.000000000	0.552590002 T T T
0.500000000	-0.000000000	0.661869998 T T T
0.000000000	0.000000000	0.279379990 T T T
-0.000000000	-0.000000000	0.388660004 F F F
0.000000000	0.000000000	0.497949986 F F F
0.000000000	0.000000000	0.607229982 T T T
0.250000000	0.250000000	0.735559998 T T T
0.250000000	0.250000000	0.783900053 T T T

POSCAR 3:

Al_2Cu(0\0\1)-B-bridge

1.0

4.1051998138	0.0000000000	0.0000000000
0.0000000000	4.1051998138	0.0000000000
0.0000000000	0.0000000000	23.5652999878

Al	Cu	B	N
8	5	1	1

Direct

0.000000000	0.500000000	0.231089990 T T T
-0.000000000	0.500000000	0.353780015 F F F
-0.000000000	0.500000000	0.476480016 F F F
-0.000000000	0.500000000	0.599170021 T T T
0.500000000	0.000000000	0.231089990 T T T
0.500000000	-0.000000000	0.353780015 F F F
0.500000000	-0.000000000	0.476480016 F F F
0.500000000	-0.000000000	0.599170021 T T T
-0.000000000	-0.000000000	0.169740010 T T T
0.000000000	0.000000000	0.292439990 T T T
-0.000000000	-0.000000000	0.415129975 F F F
-0.000000000	-0.000000000	0.537819980 T T T
0.000000000	0.000000000	0.660520021 T T T
0.500000000	-0.000000000	0.739019995 T T T
0.500000000	-0.000000000	0.793299946 T T T

POSCAR 4:

Al_2Cu(0\0\1)-N-bridge

1.0

4.1051998138	0.0000000000	0.0000000000
0.0000000000	4.1051998138	0.0000000000
0.0000000000	0.0000000000	23.5652999878

Al	Cu	N	B
8	5	1	1

Direct

0.000000000	0.500000000	0.231089990 T T T
-0.000000000	0.500000000	0.353780015 F F F
-0.000000000	0.500000000	0.476480016 F F F
-0.000000000	0.500000000	0.599170021 T T T
0.500000000	0.000000000	0.231089990 T T T
0.500000000	-0.000000000	0.353780015 F F F
0.500000000	-0.000000000	0.476480016 F F F
0.500000000	-0.000000000	0.599170021 T T T
-0.000000000	-0.000000000	0.169740010 T T T
0.000000000	0.000000000	0.292439990 T T T
-0.000000000	-0.000000000	0.415129975 F F F
-0.000000000	-0.000000000	0.537819980 T T T
0.000000000	0.000000000	0.660520021 T T T
0.500000000	-0.000000000	0.739019995 T T T
0.500000000	-0.000000000	0.793299946 T T T
